# Macrophages Mediate Mesoscale Brain Mechanical Homeostasis

**DOI:** 10.1002/adma.202517493

**Published:** 2025-11-18

**Authors:** Woong Young So, Bailey Johnson, Patricia B. Gordon, Kevin S. Bishop, Hyeyeon Gong, Hannah A Burr, Jack Rory Staunton, Chenchen Handler, Khanh Loan Ly, Raman Sood, Giuliano Scarcelli, Kandice Tanner

**Affiliations:** ^1^ Laboratory of Cell Biology Center for Cancer Research National Cancer Institute National Institutes of Health Bethesda MD 20892 USA; ^2^ Zebrafish Core National Human Genome Research Institute National Institutes of Health Bethesda MD 20892 USA; ^3^ Fischell Department of Bioengineering Maryland Biophysics Program University of Maryland College Park MD 20742 USA

**Keywords:** CSF1R, Brillouin microscopy, macrophages, optical tweezer, tissue mechanics

## Abstract

Mechanical cues orchestrate cellular behavior in the brain, yet how they interface with immune regulation remains unresolved. Here, gigahertz‐frequency Brillouin microscopy with optical trap‐based active microrheology is integrated to map the mechanical impact of macrophage activity across timescales. It is revealed that microglia, brain's resident macrophages, actively sustain tissue viscoelasticity, rather than quiescently residing within the tissues under normal conditions. Moreover, macrophage colony‐stimulating factor 1 receptor (csf1r)‐driven stromal remodeling alters brain mechanical properties in vivo. These findings establish a direct link between immune activity, viscoelastic integrity, and structural plasticity of neural tissue. By bridging high‐frequency material responses with immune‐driven remodeling, the approach provides a framework for decoding how biophysical properties regulate slower‐timescale signaling and offers mechanistic insights for increasing the efficacy of csf1r‐targeted therapies in cancer and neurodegeneration.

## Graphical abstract

We investigated the role of infiltrating immune cells in sculpting the developing brain. Using Brillouin microscopy and active microrheology, we found microglia actively maintain tissue viscoelasticity. Moreover, colony stimulating factor one receptor (CSF1R)‐driven stromal remodeling alters brain mechanics in vivo. These findings link immune activity, viscoelastic integrity, and neural tissue plasticity, offering insights for CSF1R‐targeted therapies in cancer and neurodegeneration.

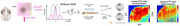

## 1 Introduction

Organ homeostasis relies on the dynamic rearrangement, expansion, and remodeling of interconnected tissues. In the brain, this involves the cooperative function of neuronal networks, specialized vascular systems like the blood–brain barrier, meningeal lymphatics, and brain‐specific immune cells, such as microglia.^[^ ^1^, ^2^, ^3^, ^4^ ^]^ In particular, microglia play an important role in the developing brain. They sculpt neuronal and astrocytic extensions to establish and maintain the neuronal connectivity essential for normal brain function.^[^ ^5^ ^]^ Their influence extends beyond development, impacting adult brain function as well.^[^ ^6^ ^]^ Recent studies indicate that impairments in these processes can contribute to the onset of pathologies like cancers and neurodegenerative diseases.^[^ ^7^, ^8^, ^9^ ^]^ Therefore, a more refined understanding of microglial dynamics and their multifaceted roles in maintaining homeostasis and contributing to disease states is critical for optimizing diagnosis and therapy in neuropathological conditions.^[^ ^10^, ^11^ ^]^ Building on D'Arcy Thompson's initial framework, modern techniques have solidified the understanding that biophysical properties, specifically tissue viscoelasticity, are crucial during development.^[^ ^12^, ^13^ ^]^ Tissue viscoelasticity influences cell migration, cell signaling, proliferation, and cell death.^[^ ^14^, ^15^ ^]^ While microglia exhibit durotaxis and can become activated by external physical cues, the influence of tissue viscoelasticity on microglial function, and whether microglia regulate tissue viscoelasticity in vivo, remains largely unexplored.^[^ ^16^, ^17^ ^]^

Viscoelasticity, a valuable measure of mechanical properties, describes a material's nonlinear response to applied forces and deformations.^[^ ^18^ ^]^ It is characterized by various moduli that reflect a material's properties under different modes (axes) of applied stresses and strains.^[^ ^18^ ^]^ These moduli encompass the combinatorial solid‐like versus liquid‐like behavior of these materials.^[^ ^18^ ^]^ Various techniques exist to measure shear, Young's, or longitudinal moduli across different scales of length, frequency, and force.^[^ ^19^ ^]^ These measurements are interchangeable, if additional parameters are known.^[^ ^19^ ^]^ For instance, the shear modulus can be calculated from the Young's modulus if the Poisson's ratio (the ratio of transverse to axial strain) is available.^[^ ^19^ ^]^ This relationship holds true for isotropic, homogeneous materials. However, living tissues are inherently anisotropic due to optical and material inhomogeneities, which arise from variations in cell types, fluids (such as blood, cerebrospinal fluid, and lymph), and specialized extracellular matrices (ECM) within organs.^[^ ^20^ ^]^ Therefore, additional and often empirical information is necessary to fully characterize the material response of living tissues. To comprehensively understand the frequency response of viscoelastic materials, here we compared measurements from established methods across several decades of frequencies: (10–1000s of Hz and gigahertz frequencies). However, noninvasively mapping of tissue mechanical properties at a resolution sufficient for interpreting cellular processes and signaling remains a challenge.

Here, our studies in zebrafish focused on optically probing brain morphogenesis to decipher the interplay between mechanical signatures and microglial dynamics. The zebrafish is a widely used model organism due to the conservation of many organs with mammalian counterparts and the transparency of larval zebrafish, which enables noninvasive optical techniques.^[^ ^21^, ^22^, ^23^, ^24^, ^25^, ^26^ ^]^ We utilized two independent microscale mechanical mapping methods: Optical tweezer‐based active microrheology (OT‐AMR) and Brillouin microscopy. A critical distinction lies in the temporal scales of optical trapping (OT‐AMR) measurements, compared to those of Brillouin spectroscopy.^[^ ^24^, ^27^, ^28^, ^29^ ^]^ Specifically, OT‐AMR probes material mechanical properties and dynamics at a much lower frequency regime than Brillouin microscopy. This disparity in temporal scale significantly impacts the comparison of data from both techniques, as they are sensitive to different aspects of material behavior. To address this, we converted the Brillouin shift to Longitudinal Modulus using experimentally derived values for tissue density. We then compared the microscale shear and longitudinal moduli for each of the modalities to map development for the same animal over days. With these in hand, we aimed to explore the complete material response. We then investigated the role of mechanically mediated tissue remodeling in larval zebrafish by employing a suite of tools to chemically or genetically ablate macrophages. We uncovered that the tissue viscoelasticity was influenced by both macrophages and colony stimulating factor 1 receptor receptor‐mediated crosstalk within the developing brain. These results were further supported by studies using brain‐mimetic hydrogels, in which mammalian and zebrafish microglia were embedded at various densities. Additionally, we established that macrophage activity was modulated by changes in ECM stiffness. Our data suggest that the viscoelastic properties of tissue are linked to macrophage‐mediated tensional homeostasis in the brain. Furthermore, these findings imply that macrophage dynamics, which induce acute mechanical changes, may play an increasingly significant role in alterations to normal tissue architecture and in therapies targeting these changes.

## 2 Main

### 2.1 Optical Trap Active Microrheology and Brillouin Microscopy Show Strong Correlation for Measured Microscale Mechanics of Functionally Distinct Units of the Brain

**Figure 1:**
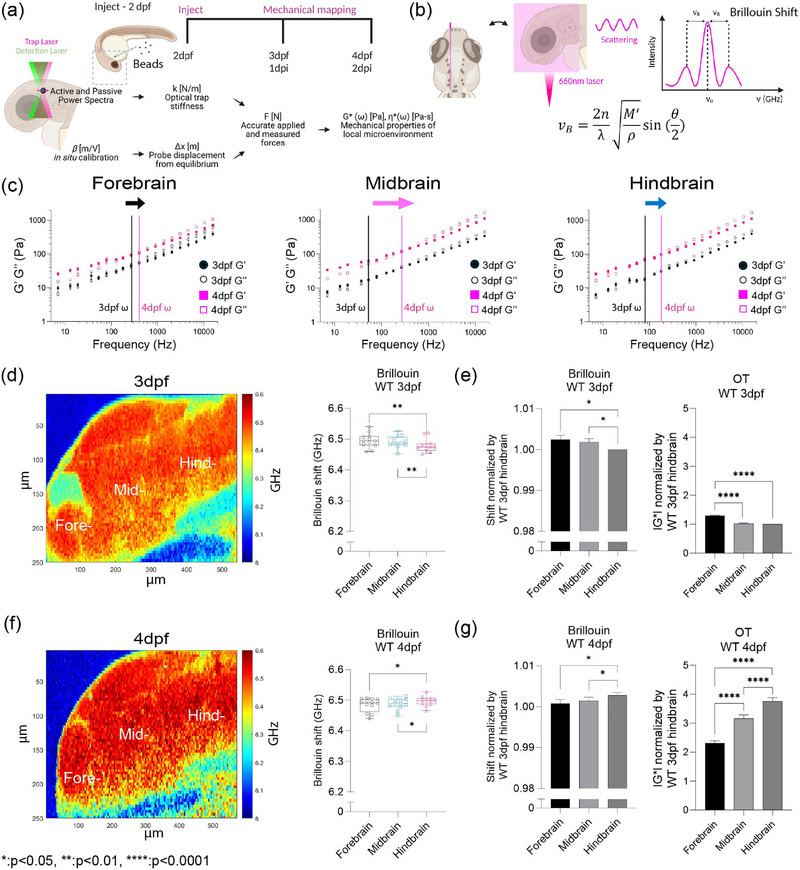
Optical trap active microrheology, Brillouin microscopy show similar trends for measured microscale mechanics of functionally distinct units of the brain. a) Schematic of brain mechanical mapping using optical trap based active microrheology (OT‐AMR) where 1 µm beads are injected into midbrain parenchyma at 3 days postfertilization (3 dpf)/1 day postinjection (dpi), 4 dpf/2 dpi. These beads served as mechanical probes where a focused beam using a high‐power trap laser oscillates the bead, while a stationary low‐power detection laser records the bead motion, which is translated into complex modulus (|G*|), elastic modulus (*G*′), viscous modulus (*G*″) using the appropriate calibration steps. b) Schematic of the experimental setup where the Brillouin shift (*v*_B_) is determined by measuring the inelastic scattered light from the brain parenchyma following excitation with 660 nm laser radiation. The equation shows the relationship between Brillouin shift, Longitudinal Modulus, collection angle of scattered light, laser excitation wavelength, density, refractive index. c) Log–log plots showing the comparison of broadband values obtained using OT‐AMR of wild‐type (WT) brain mechanical properties (elastic modulus, *G*′, viscous modulus, *G*″) for the frequency range from 7 Hz to 15 kHz, as a function of developmental stage (3 dpf, 4 dpf), brain regions (forebrain, midbrain, hindbrain). The crossover frequency where viscous modulus becomes greater than elastic modulus is labeled as follows: 3 dpf in black, 4 dpf in magenta for each brain region. Error bars represent the standard error for each measurement (3 dpf = 4 fish‐forebrain *n* (number of beads/fish) = 78, midbrain *n* = 90, hindbrain *n* = 77. 4 dpf = 6 fish – forebrain *n* (number of beads/fish) = 202, midbrain *n* = 224, hindbrain *n* = 259). d) A representative micrograph depicts an image of Brillouin shifts of a 3 dpf wild‐type (WT) zebrafish brain where forebrain, midbrain, hindbrain regions are labeled. The heat map indicates Brillouin shift per pixel. The scale represents color‐coded Brillouin shift where red indicates increased shift, blue indicates lower shift (Left panel). The graph shows the average Brillouin shifts (GHz) for each region (“Fore‐,” “Mid‐,” “Hind‐”) for *n* = 12 zebrafish. **p < 0.01, paired two‐tailed *t*‐tests; confidence interval = 95%. e) Bar graph of normalized Brillouin shifts of wild‐type 3 dpf with respect to the average Brillouin shift measured from the wild‐type 3 dpf hindbrain (Left panel), a bar graph of normalized wild‐type 3 dpf complex modulus (|G*|) obtained using OT‐AMR with respect to wild‐type 3 dpf hindbrain averaged across all the frequencies. *p < 0.05,, ****p < 0.0001, paired two‐tailed *t*‐tests; confidence interval = 95%. f) A representative micrograph depicts an image of Brillouin shifts of a 4 dpf wild‐type (WT) zebrafish brain where forebrain, midbrain, hindbrain regions are labeled. The heat map indicates Brillouin shift per pixel. The scale represents color‐coded Brillouin shift where red indicates increased shift, blue indicates lower shift (Left panel). The graph shows the average Brillouin shifts (GHz) for each region (“Fore‐,” “Mid‐,” “Hind‐”) for *n* = 12 zebrafish. *p < 0.05, paired two‐tailed *t*‐tests; confidence interval = 95%. g) Bar graph of normalized Brillouin shifts of wild‐type 4 dpf with respect to the average Brillouin shift measured from the wild‐type 3 dpf hindbrain (Left panel), a bar graph of normalized wild‐type 3 dpf complex modulus (|G*|) obtained using OT‐AMR with respect to wild‐type 3 dpf hindbrain averaged across all the frequencies. *p < 0.05, ****p < 0.0001, paired two‐tailed *t*‐tests; confidence interval = 95%.

The formation of the forebrain, midbrain, hindbrain, and blood–brain barrier in both zebrafish and mammals is a conserved developmental process (Figure S1a, Supporting Information).^[^ ^30^, ^31^ ^]^ We reasoned that there would be regional differences in mechanical properties, and this should change as a function of development (Figure S1a, Supporting Information). To measure the mechanical properties of the developing brain from 3 to 4 post‐fertilization (dpf), we employed optical trap based active microrheology (OT‐AMR) (**Figure** **1**a,c; and Figure S1b–e, Supporting Information). At 2 dpf, we introduced 1‐µm‐diameter polystyrene spheres directly into the brain parenchyma. These spheres served as mechanical probes, to which we applied a multiplexed sinusoidal oscillation with an amplitude of a total of 200 nm, across a frequency range of 7 Hz to 15 kHz.^[^ ^24^ ^]^ Bead displacements were simultaneously tracked using back focal plane interferometry.^[^ ^24^ ^]^ Using a vis‐a‐vis calibration and detection beam steering method, quantitation of the local viscoelasticity, G*(*ω*) at that bead position is achieved by correlating the trap and bead positions.^[^ ^22^, ^32^ ^]^ The complex modulus, G*(*ω*) = |G*|exp(iδ) = *G*′(*ω*) +*iG*″(*ω*) which then gives storage (*G*′(*ω*)) and loss (*G*″(*ω*) moduli). Then, comparing *G*′ (elastic modulus) and *G*″ (viscous modulus) by *G*″/*G*′ would result into loss tangent or hysteresivity to determine the relative state between solid‐like (more *G*′ contribution) or liquid‐like characteristics (more *G*″ contribution) based on crossover frequency. We show that brain tissue is mechanically heterogeneous as a function of region of the brain and age (Figure 1c; and Figure S1b–e, Supporting Information). At 3 dpf, the midbrain is more liquid‐like than the hind‐ and forebrain, respectively. In contrast, the forebrain is more rigid than the mid‐ and hindbrain. As the brain develops, all regions become more solid‐like with the greatest transition occurring for the midbrain. An increase in the complex modulus is observed for all regions with the hind brain showing the greatest increase. We then asked if our measured viscoelasticity (complex moduli (G*)) obeyed frequency‐dependent power laws, |G*(*ω*)| = *aω* ^b^, where the dependence b varies for different frequency regimes and different cell types.^[^ ^29^, ^33^, ^34^ ^]^ Here, *G*′ and *G*″ monotonically increase as a function of frequency and show power law dependence at frequencies >400 Hz (Figure 1c; and Figure S1b,c, Supporting Information). Using these metrics, regional comparison at 3 dpf showed that the slope of the line is greatest for the hindbrain (0.600) followed by the forebrain (0.582) and then the midbrain (0.565) where each of the values fall between 0.5 and 0.75 which correspond to formulaic descriptions of flexible and semiflexible polymers, respectively (Figure S1b,c, Supporting Information). As the animal ages, the midbrain shows the greatest change where the slope of the line now changes from 0.565 to 0.607 (Figure S1b,c, Supporting Information) along with increase of macrophage population (Figure S1a, Supporting Information). The normalized complex modulus data from whole frequency range by 3 dpf hindbrain shows that each brain regions has distinct mechanical property with statistical significance based on paired *t*‐test (Figure S1b, Supporting Information). All regions stiffen as a function of age but not uniformly where the hindbrain shows the greatest increase in stiffness (Figure S1d,e, Supporting Information).

OT‐AMR offers the advantage of quantifying absolute values of viscoelasticity.^[^ ^22^, ^24^ ^]^ However, the technique necessitates the introduction of probes, that if introduced in sufficient numbers can perturb normal physiology. Mechanical phenotypes of large tissues can only be obtained by averaging across many samples assuming random bead distribution. Thus, assessing the viscoelasticity of an individual fish is difficult. Brillouin microscopy (BM) can overcome this challenge as it is a purely noninvasive technique, based on the intrinsic inelastic scattering due to incident photon–phonon interactions in materials, whose spectral characterization provides information correlated with the underlying mechanical properties (Figure 1b).^[^ ^28^, ^35^ ^]^ Formulaically, the frequency shift (*v*_B_) of Brillouin scattered photons (on the order of GHz) relate to the local refractive index (*n*), mass density (𝜌), and the longitudinal elastic modulus (*M*′) of the material of interest (Figure 1b).^[^ ^28^, ^35^ ^]^ Thus, stiffer samples would result in higher frequency shifts. We determined that Brillouin shifts were comparable in detecting mechanical heterogeneity of brain regions at 3 and 4 dpf (Figure 1d,f). Furthermore, the trends in stiffness at 3 and 4 dpf based on Brillouin microscopy are the same as those measured using OT‐AMR (Figure 1d–g). At 3 dpf, hindbrain is the softest but becomes the stiffest at 4 dpf with a significant increase in frequency shift (Figure 1e,g; and Figure S1f, Supporting Information).

### 2.2 Brillouin Longitudinal Modulus Using Calculated Density Factor Shows Greater Correlation for OT‐AMR Measurements Obtained at Lower Frequencies in the Zebrafish Brain

**Figure 2:**
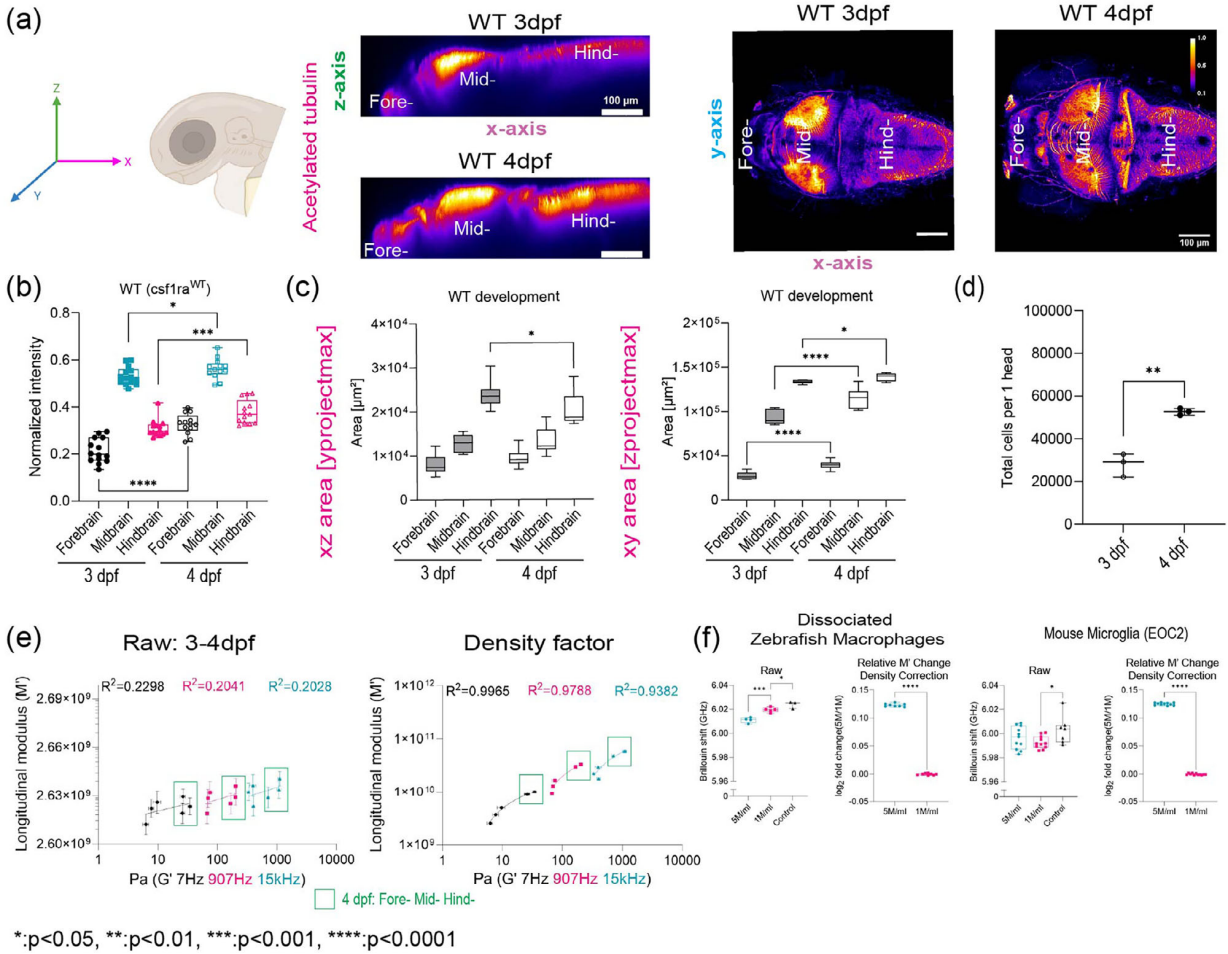
Brillouin longitudinal modulus using calculated density factor shows greater correlation for OT‐AMR measurements obtained at lower frequencies in vivo. a) Schematic of immunofluorescence of acetylated tubulin in different axis projections; *z*‐axis, *y*‐axis focusing on brain parenchyma excluding eye, *y*‐axis maximum projection of wild type (WT) acetylated tubulin over the development, *z*‐axis maximum projection of wild type acetylated tubulin intensity excluding eye over the development. b) Quantitation of normalized acetylated tubulin intensity in terms of development, brain regions (wild type 3 dpf: *n* = 14, wild type 4 dpf: *n* = 13). *p < 0.05, ***p < 0.001, ****p < 0.0001, unpaired two‐tailed *t*‐tests; confidence interval = 95%. c) Quantitation of brain region area based on acetylated tubulin in terms of development, xz (*y*‐axis maximum projection), xy (*z*‐axis maximum projection) (wild type 3 dpf: *n* = 14, wild type 4 dpf: *n* = 13). *p < 0.05, ****p < 0.0001, unpaired two‐tailed *t*‐tests; confidence interval = 95%. d) Dissociated head cells per fish based on the cluster of 15 fish in terms of development (wild type 3 dpf: *n* = 45, wild type 4 dpf: *n* = 45). Each dot represents a group of 15 fish head. **p < 0.01, unpaired two‐tailed *t*‐tests; confidence interval = 95%. e) Raw correlation between corrected Brillouin (longitudinal) modulus (*M*′), OT‐AMR elastic modulus (*G*′) at 7 Hz, 907 Hz, 15 kHz during development (3–4 dpf) in log–log plot in comparison with density factor corrected correlation between corrected Brillouin (longitudinal) modulus (*M*′), OT‐AMR elastic modulus (*G*′) at 7 Hz, 907 Hz, 15 kHz during development (3–4 dpf) in log–log plot. f) Raw Brillouin shift of hyaluronic acid gel mechanical remodeling at different cell densities of 5 million cells mL^−1^ (*n* = 4), 1 million cells mL^−1^ (*n* = 5), control (*n* = 3) (no cells) for dissociated zebrafish macrophages (Mpeg1.1+) from zebrafish head, along with relative change in density corrected longitudinal modulus (*M*′) in log_2_(fold change). Raw Brillouin shift of hyaluronic acid gel mechanical remodeling at different cell densities of 5 million cells mL^−1^ (*n* = 11), 1 million cells mL^−1^ (*n* = 11), control (*n* = 7) (no cells) for mouse microglia (EOC2), along with relative change in density corrected longitudinal modulus (*M*′) in log_2_(fold change). *p < 0.05, ***p < 0.001, ****p < 0.0001, unpaired two‐tailed *t*‐tests; confidence interval = 95%.

We then aimed to link tissue architecture to the measured mechanical phenotype (**Figure** **2**a). One of the most dramatic changes observed is that the neuronal networks increase in size and complexity as the brain develops. Recently, it has been proposed that the acetylation of lysin 40 of α‐tubulin, a key component of neurons, is important for stiffness of developing tissue as well as cell mechanics.^[^ ^36^, ^37^ ^]^ Whole‐mount immunofluorescence of acetylated tubulin of the brain shows differential structure and clustering of neuronal networks between 3 and 4 dpf, especially in the midbrain and hindbrain. An increase in normalized intensity of acetylated tubulin was observed for all brain regions during development (Figure 2a,b). BM has been recently used to noninvasively infer the mechanical properties of engineered thick tissues, such as organoids and in living animals.^[^ ^28^, ^35^, ^38^ ^]^ Converting the measured Brillouin shift into an absolute longitudinal modulus requires knowledge of additional optical and material properties such as the mass density and refractive index.^[^ ^39^, ^40^ ^]^ Assessing these parameters in vivo is challenging due to the inherent inhomogeneities of tissue. To reconcile these differences, in this study we attempted to quantify density by counting the number of cells for each of the brain regions, forebrain, midbrain, and hindbrain by dissociating these tissues (Figure 2c,d). We then used the areas calculated from imaging to provide an estimate for the density correction. These calculated values were then applied after a constant value for mass density for all regions, using previously published values of 1.04 g mL^−1^ density and the refractive index of 1.35 for a HeLa cell.^[^ ^41^ ^]^ Here, we refer to the Longitudinal modulus, *M*′, using the values obtained from HeLa cells as “Raw” *M*′. These values were used for all the regions. To address the disparity between the timescales of the two methods, we plotted the values as a function of those obtained for the *G*′ at the frequencies of 7, 907, and 15 kHz measured using OT‐AMR (Figure 2e, left panel; and Figure S2a, Supporting Information). We then compared the raw and corrected correlation between Brillouin (longitudinal) modulus, *M*′ and OT‐AMR at 7 Hz, 907 Hz, and 15 kHz in terms of the elastic modulus *G*′, for development stages during 3–4 dpf (Figure 2e; and Figure S2a,b, Supporting Information). Comparison of linear fits between log (*G*′) for forebrain, midbrain, and hindbrain) and log(calculated Brillouin modulus *M*′) revealed poor correlation between techniques before corrections based on cell line‐based refractive index and density (Figure 2e; and Supporting Information S2). While the overall correlations improved postcorrection, better fits were obtained for values measured at 7 and 907 Hz compared to 15 kHz (Figure 2e; and Figure S2a,b, Supporting Information). In addition to an increase in resident cell populations, development is characterized by the infiltration of various cell types, including macrophages (Figure 2d). We next quantified mid‐brain single plane macrophage populations throughout development (Figure 2d; and Figure S1a, Supporting Information). To assess the influence of these infiltrating cells on mechanical properties, we embedded isolated microglia into brain‐like extracellular matrix (hyaluronic acid) hydrogels. We compared the impact of macrophage density on brain‐mimetic hydrogels on measured mechanical properties. Control hydrogels exhibited the most substantial BM shift when compared to those with both low and high macrophage densities (Figure 2f). Following density correction, hydrogels with elevated macrophage densities demonstrated increased modulus. These findings were consistently observed in isolated zebrafish microglia and a mouse microglial cell line (EOC2) embedded in a 3D Hyaluronic (HA) gel (Figure 2f).

### 2.3 OT‐AMR and Brillouin Microscopy can Detect Changes in Tissue Mechanical Properties in Csf1r Zebrafish Mutant with Delayed Macrophage Invasion

**Figure 3:**
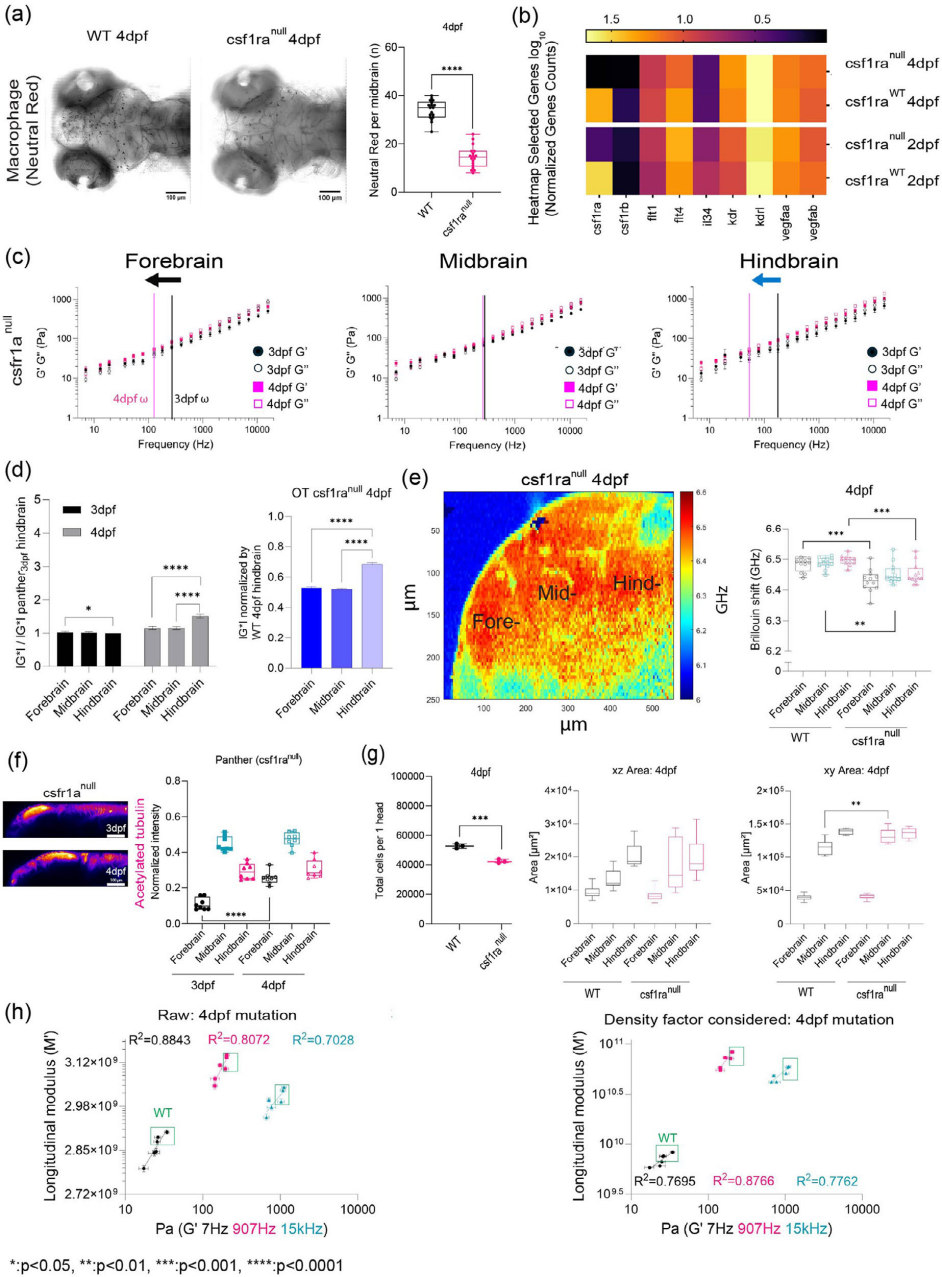
OT‐AMR, Brillouin microscopy measured distinct mechanical differences in tissue mechanical properties in a zebrafish mutant characterized by impaired macrophage invasion. a) Micrographs showing macrophages located in one z plane in the midbrain as labeled by neutral red assay comparing between wild type, *panther* (*csf1ra* ^null^) fish at 4 dpf (wild type 4 dpf: *n* = 18, *csf1ra* ^null^ 4 dpf: *n* = 18). ****p < 0.0001, unpaired two‐tailed *t*‐tests; confidence interval = 95%. b) Heatmap of a subset of genes from bulk RNA‐Sequencing at 2 dpf, 4 dpf comparing *csf1ra* mutant to wild type fish. c) Log–log plots showing the comparison of broadband values obtained using OT‐AMR of wild‐type (WT) brain mechanical properties (elastic modulus, *G*′, viscous modulus, *G*″) for the frequency range from 7 Hz to 15 kHz, as a function of developmental stage (3 dpf, 4 dpf), brain regions (forebrain, midbrain,, hindbrain). The crossover frequency where viscous modulus becomes greater than elastic modulus is labeled as follows: 3 dpf in black, 4 dpf in magenta for each brain region. Error bars represent the standard error for each measurement (3 dpf = 4 fish – forebrain *n* (number of beads/fish) = 65, midbrain *n* = 88, hindbrain *n* = 73. 4 dpf = 5 fish – forebrain *n* (number of beads/fish) = 141, midbrain *n* = 140, hindbrain *n* = 168). d) A bar graph of normalized 3, 4 dpf *csf1ra* ^null^ complex modulus (|G*|) obtained using OT‐AMR with respect to 3 dpf *csf1ra* ^null^ hindbrain averaged across all the frequencies (*n* = 19), *p < 0.05, ****p < 0.0001, paired two‐tailed *t*‐test; confidence interval = 95% (Left panel). A bar graph of normalized 4 dpf *csf1ra* ^null^ complex modulus (|G*|) obtained using OT‐AMR with respect to 4 dpf wild‐type hindbrain averaged across all the frequencies (*n* = 19) to show the degree of increase compared to c*sf1ra* ^null^. (*n* = 19), ****p < 0.0001, paired two‐tailed *t*‐test; confidence interval = 95% (Right panel). e) A representative micrograph depicts an image of Brillouin shifts of a 4 dpf *csf1ra* ^null^ zebrafish brain where forebrain, midbrain, hindbrain regions are labeled. The heat map indicates Brillouin shift per pixel. The scale represents color‐coded Brillouin shift where red indicates increased shift, blue indicates lower shift (Left panel). The graph shows the average Brillouin shifts (GHz) for each region (“Fore‐,” “Mid‐,” “Hind‐”), comparing with wild type (wild type = 12, *csf1ra* ^null^ = 12). **p < 0.01, ***p < 0.001, unpaired two‐tailed *t*‐tests; confidence interval = 95%. f) *y*‐axis maximum projection of *csf1ra* ^null^ acetylated tubulin over the development, its normalized intensity in terms of development, brain regions (3 dpf *n* = 9, 4 dpf *n* = 7). g) Dissociated head cells per fish based on the cluster of 15 fish comparing *csf1ra* mutation to the wild type at 4 dpf (wild type 4dpf: *n* = 45, *csf1ra* ^null^ 4dpf: *n* = 45). Each dot represents a group of 15 fish head. ***p < 0.001, unpaired two‐tailed *t*‐tests; confidence interval = 95%. Quantitation of brain region area based on acetylated tubulin in terms of *csf1ra* mutation at 4 dpf, xz (*y*‐axis maximum projection), xy (*z*‐axis maximum projection) (wild type 4 dpf *n* = 13, *csf1ra* ^null^ *n* = 7). **p < 0.01, unpaired two‐tailed *t*‐tests; confidence interval = 95%. h) Raw correlation between corrected Brillouin (longitudinal) modulus (*M*′), OT‐AMR elastic modulus (*G*′) at 7 Hz, 907 Hz, 15 kHz at *csf1ra* mutation at 4 dpf in log–log plot along with density factor corrected correlation between corrected Brillouin (longitudinal) modulus (*M*′), OT‐AMR elastic modulus (*G*′) at 7 Hz, 907 Hz, 15 kHz at *csf1ra* mutation at 4 dpf in log–log plot.

To assess if both the presence and function of microglia regulated tissue mechanics, we employed the zebrafish mutant, *panther*, a mutant which lacks a functional *csf1ra* (*fms*, M‐CSF receptor ortholog) gene.^[^ ^42^ ^]^ Colony stimulating factor 1 receptor (*csf1ra*) is a key determinant in microglia development.^[^ ^43^, ^44^, ^45^ ^]^ In these fish, early macrophages differentiate and behave normally in the yolk sac,^[^ ^45^ ^]^ however, there is a delayed invasion of macrophages into the brain. Colonization of the larval brain is eventually achieved with ≈4‐day delay.^[^ ^45^ ^]^ We determined that the mutant showed a difference in brain morphology and gene expression at 4 dpf compared to WT (**Figure** **3**a,b; and Figure S3a,b, Supporting Information). Furthermore, there are decreased numbers of macrophages in the mutant brain compared to the wild type (Figure 3a). Additionally, the blood–brain barrier (BBB) is impaired in the mutant fish where increased leakage of 150 kDa dextran is observed (Figure S3b, Supporting Information) compared to wild type (Figure S1a, Supporting Information). OT‐AMR measurements revealed that the regional heterogeneity observed in wild type is lost where the magnitude of the complex modulus is comparable for all regions (Figure 3c,d). Direct comparison to wild type revealed that the mutant brain is softer in all brain regions at 4 dpf (Figure 3e). Additionally, the fore‐ and hindbrain in the mutant become more liquid‐like during development as the crossover frequency where viscous modulus (*G*″) crosses over elastic modulus (*G*′) shifts to lower frequency (Figure 3c). G*, *G*′, and *G*″ monotonically increase as a function of frequency. At frequencies exceeding 400 Hz, a power law dependence was observed (Figure 3c; and Supporting Information 3c,d). Conversely, the midbrain's mechanical properties exhibited a modest developmental increase, accompanied by minimal changes in the power law exponent (Supporting Information 3c–f). Analysis of the normalized complex modulus across the entire frequency range in 3 dpf hindbrain indicated a more mechanically homogeneous brain (Figure 3d). Brillouin microscopy similarly revealed reduced Brillouin shifts in the mutant compared to wild type across all brain regions at 4 dpf (Figure 3e). In addition to quantitation of fewer macrophages, acetylated tubulin staining in the *panther* mutant showed greater homogeneity during development compared to wild type (Figures 2a,b and 3f). Following the methodology applied for wild type fish, cells were isolated from each region of the mutant fish to calculate the density. Subsequent calculations compared the raw and corrected correlations between Brillouin (longitudinal) modulus (*M*′) and OT‐AMR elastic modulus (*G*′) at 7 Hz, 907 Hz, and 15 kHz, expressed as the elastic modulus at the 4 dpf developmental stage for both wild type and mutant zebrafish (Figure 3g,h; and Figure S4a,b, Supporting Information). Comparison of linear fits between log(*G*′) for forebrain, midbrain, and hindbrain) and log (calculated Brillouin modulus *M*′) revealed poor correlation between techniques before corrections based on estimated brain refractive index and density (Figure 3h; and Figure S4a,b, Supporting Information). The overall correlations improved post correction for the measurements of 907 Hz and 15 kHz but no significant change for the measurements at 7 Hz (Figure 3h; and Figures S4a,b and S5a–e, Supporting Information). However, the fits were relatively low compared to the fits obtained post correction for the wild type fish as a function of development (Figure 2e; and Figure S2a,b, Supporting Information).

### 2.4 Csf1r Macrophages Density Regulates Brain Mechanics

**Figure 4:**
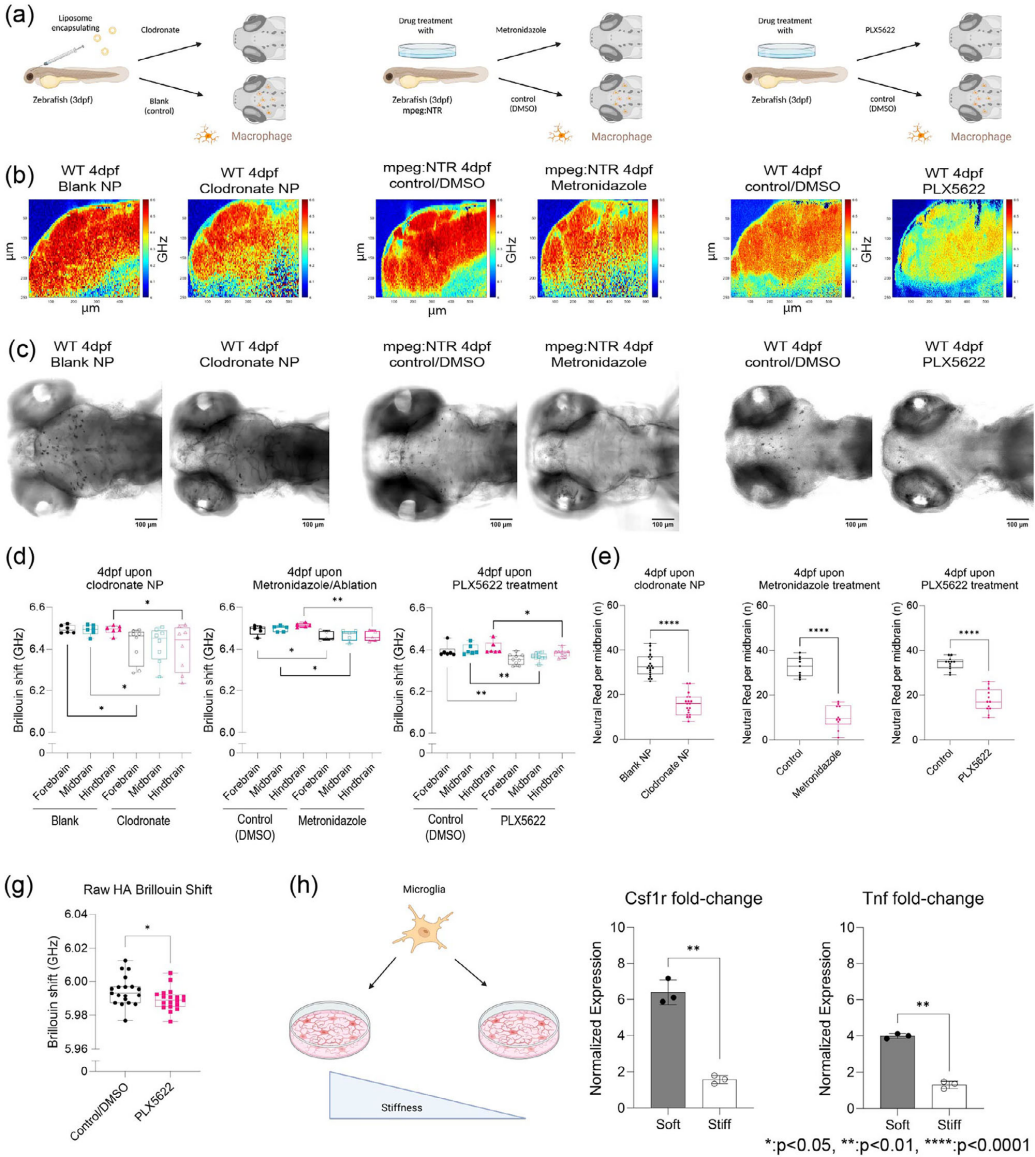
Macrophages, Csf1r‐mediated stromal interactions regulate brain mechanics. a) Schematic describing macrophage depletion using clodronate. Briefly, nanoparticles (liposomes encapsulating clodronate) were injected into wild type 3 dpf midbrain (Left panel). Schematic describing macrophage depletion using chemogenetics where *mpeg1.1:NTR* fish were treated with Metronidazole, all mpeg macrophages would be eliminated (Center panel). Schematic of CSF1R inhibition using small molecule drug, PLX5622 on wild type at 3 dpf (Right panel). b) A representative micrograph depicts an image of Brillouin shifts of a 4 dpf wild type zebrafish brain upon blank nanoparticle (NP), clodronate NP injection to deplete macrophage (Left panel). A representative micrograph depicts an image of Brillouin shifts of a 4 dpf *mpeg1.1:NTR* fish upon Metronidazole treatment to ablate macrophages (control/DMSO vs Metronidazole) (Center panel). A representative micrograph depicts an image of Brillouin shifts of a 4 dpf wild type fish upon PLX5622 treatment to inhibit CSF1R (control/DMSO vs Metronidazole) (Right panel). The heat map indicates Brillouin shift per pixel. The scale represents color‐coded Brillouin shift where red indicates increased shift, blue indicates lower shift. c) Neutral red staining images of macrophage depletion upon clodronate encapsulating liposome injection into brain, macrophage ablation upon metronidazole treatment, CSF1R inhibition upon PLX 5622 drug treatment. d) The graph shows the average Brillouin shifts (GHz) for each region (“Fore‐,” “Mid‐,” “Hind‐”) comparing with 4 dpf wild type between blank NP = 5, clodronate NP = 8 (Left panel). The graph shows the average Brillouin shifts (GHz) for each region (“Fore‐,” “Mid‐,” “Hind‐”) comparing with 4 dpf *mpeg1.1:NTR* between control/DMSO = 5, Metronidazole = 5 (Center panel). The graph shows the average Brillouin shifts (GHz) for each region (“Fore‐,” “Mid‐,” “Hind‐”) comparing with 4 dpf wild type between control/DMSO = 6, PLX5622 = 9 (Right Panel). *p < 0.05, **p < 0.01, unpaired two‐tailed *t*‐tests; confidence interval = 95%. e) Quantification of macrophages in the midbrain labeled by neutral red assay for wild type 4 dpf upon nanoparticle injection (wild type 4 dpf control NP: *n* = 20, wild type 4 dpf clodronate NP: *n* = 19). Quantification of macrophages in the midbrain labeled by neutral red assay for *mpeg1.1:NTR* 4 dpf upon Metronidazole (control NP: *n* = 9, wild type 4dpf clodronate NP: *n* = 10). Quantification of macrophages at midbrain labeled by neutral red assay for wild type 4 dpf upon PLX5622 treatment (control/DMSO: *n* = 12, PLX5622: *n* = 12). ****p < 0.0001, unpaired two‐tailed *t*‐tests; confidence interval = 95%. g) The graph shows the average Brillouin shifts (GHz) for hyaluronic acid gel mechanical remodeling by mouse microglia (EOC2) upon PLX5622 treatment (control/DMSO = 19, PLX5622 = 18). *p < 0.05, unpaired two‐tailed *t*‐tests; confidence interval = 95%. h) Schematic of mouse microglia cultured on soft, stiff substrates, followed by quantitative RT‐PCR analysis of *Csf1r*, *Tnf* mRNA expression levels. **p < 0.01, unpaired two‐tailed *t*‐tests; confidence interval = 95%.

To confirm that the presence of macrophages or their remodeling capabilities can directly perturb mechanical properties of the brain, we ablated macrophages using two methods and blocked the receptor using pharmacological methods (**Figure** **4**a). First, we injected nanoparticles (liposomes encapsulating clodronate) directly into the brain (Figure 4a–e, left panel). Second, we employed a chemogenetic model, Tg(*mpeg1.1:NTR‐IRES‐eGFP‐CAAX*)^c057^, a transgenic fish where macrophages express an enhanced variant of *Escherichia coli* nitroreductase (NfsBT41Q/N71S/F124T) (Ntr), resulting in macrophage‐specific ablation (Figure 4a–e, middle panel).^[^ ^46^ ^]^ Upon treatment with Metronidazole, Mpeg1.1^+^ cells are ablated. In each condition, we observed a reduction of macrophages. We then used a small molecule drug, PLX5622, against the Csf1r receptor to assess if macrophage‐mediated tissue remodeling is important for mechanical homeostasis (Figure 4a–e, right panel). After 1‐day postinjection (dpi) of clodronate, there was ≈55% decrease in macrophage population and ≈65% reduction using the tissue‐specific ablation based on neutral red staining assay (Figure 4c,e) compared to control fish. Macrophage depletion due to clodronate liposomes, macrophage‐specific ablation, and CSF1R inhibitor (PLX5622) caused softening of the whole brain parenchyma (Figure 4b,d) as all brain regions had a decrease in frequency shifts in the range of 70–90 MHz compared to control counterparts. The hindbrain showed the most significant reduction in stiffness concomitant with alterations in acetylated tubulin expression (Figure S6a–d, Supporting Information). We observed a decrease of macrophages in the treated fish concomitant with a softening of all brain regions as measured using BM (Figure 4b,d). However, the most significant decrease in BM shift was observed for fish with the specific genetic ablation of the macrophages. We previously extensively characterized the broadband micro and bulk rheological properties of Matrigel and hyaluronic acid hydrogels. These hydrogels at specific protein concentrations exhibited values ranging from ≈10 to 150 kPa across frequencies of 0.1 Hz to 15 kHz at different applied strains, consistent with mammalian brain mechanical properties.^[^ ^22^ ^]^ We subsequently employed these brain‐mimetic hydrogels (hyaluronic acid) to investigate the reciprocal relationship between CSF1R‐mediated mechanical modeling and macrophage activity. Cells were seeded at equal densities, with and without a CSF1R inhibitor. We observed that the vehicle control hydrogel exhibited greater stiffness compared to hydrogels where activity was inhibited (Figure 4g). Next, we examined whether gel stiffness modulated macrophage activity (Figure 4h). Our qRT‐PCR measurements revealed increased expression of both *Csf1r* and *Tnf* in mouse microglial cells cultured on softer substrates compared to those in stiffer gels (Figure 4h).

### 2.5 Modulation of Cytoskeleton of Brain Tissue is a Major Driver of Mechanical Properties and Alters Macrophage Numbers

**Figure 5:**
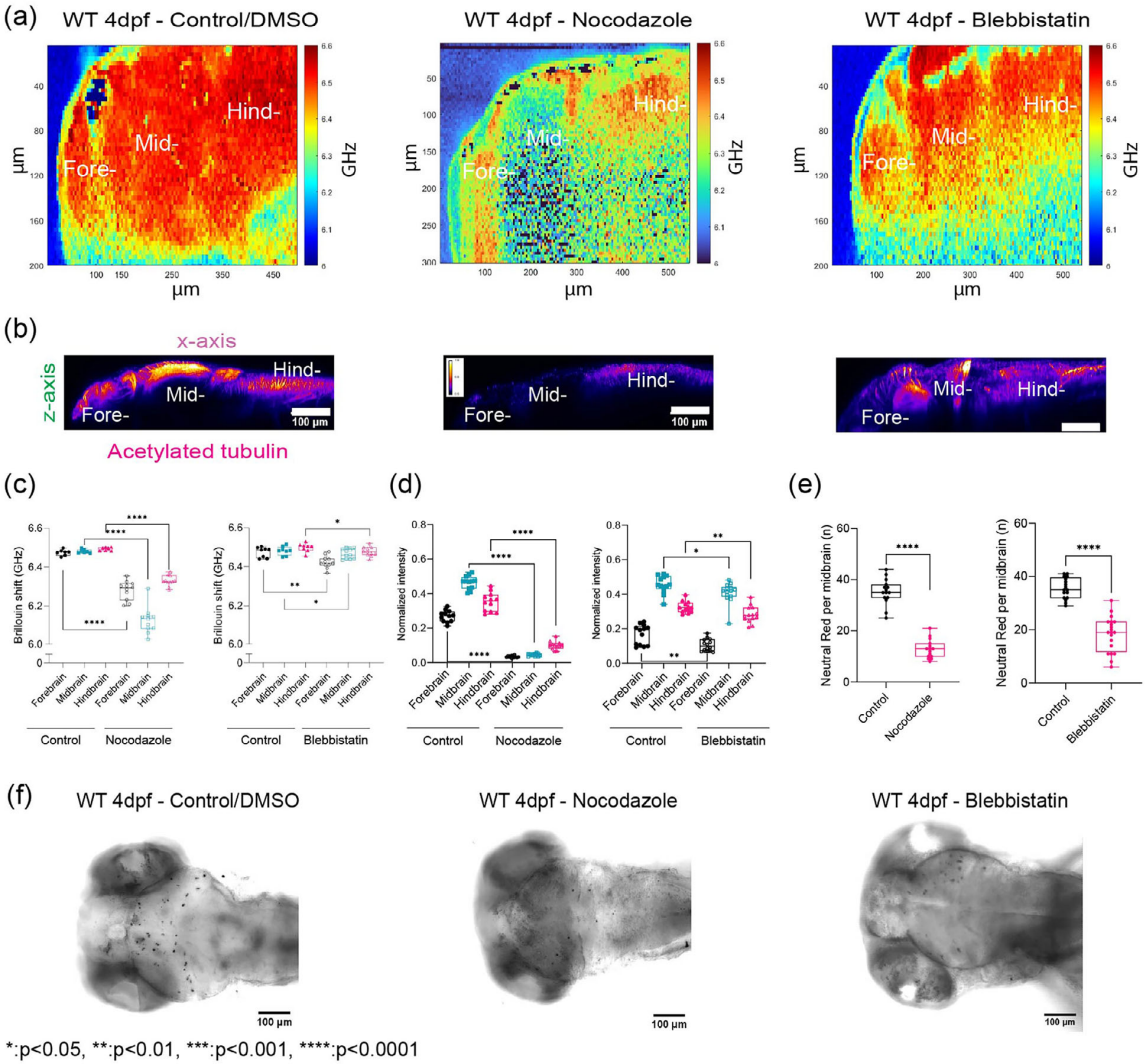
Acetylated tubulin is a major driver of brain mechanical properties. a) A representative micrograph depicts an image of Brillouin shifts of a 4 dpf wild type fish upon control/DMSO, Nocodazole treatment, Blebbistatin treatment. The heat map indicates Brillouin shift per pixel. The scale represents color‐coded Brillouin shift, where red indicates increased shift, blue indicates lower shift. b) *z*‐axis maximum projection (xy), *y*‐axis maximum projection (xz) for wild type 4 dpf acetylated tubulin of control/DMSO, Nocodazole treatment, Blebbistatin treatment with quantitation of normalized acetylated tubulin intensity for different brain regions. c) The graph shows the average Brillouin shifts (GHz) for each region (“Fore‐,” “Mid‐,” “Hind‐”) comparing with 4 dpf wild type between control/DMSO = 7, Nocodazole = 11 (Left Panel). The graph shows the average Brillouin shifts (GHz) for each region (“Fore‐,” “Mid‐,” “Hind‐”) comparing with 4 dpf wild type between control/DMSO = 5, Blebbistatin = 7 (Right Panel). *p < 0.05, **p < 0.01, ****p < 0.0001 unpaired two‐tailed *t*‐tests; confidence interval = 95%. d) Quantitation of normalized acetylated tubulin intensity for different brain regions upon Nocodazole treatment (control: *n* = 12, Nocodazole: *n* = 13). Quantitation of normalized acetylated tubulin intensity for different brain regions upon Blebbistatin treatment (control: *n* = 13, Blebbistatin: *n* = 14). *p < 0.05, **p < 0.01, ****p < 0.0001, unpaired two‐tailed *t*‐tests; confidence interval = 95%. e) Macrophages in the midbrain labeled by neutral red assay for wild type upon Nocodazole treatment (wild type 4 dpf control/DMSO: *n* = 15, wild type 4 dpf Nocodazole: *n* = 16) (Left panel). Macrophages in the midbrain labeled by neutral red assay for wild type upon Blebbistatin treatment (wild type 4 dpf control/DMSO: *n* = 17, wild type 4 dpf Blebbistatin: *n* = 20) (Right panel). ****p < 0.0001, unpaired two‐tailed *t*‐tests; confidence interval = 95%. f) Representative images of neutral red staining for 4 dpf wild type control/DMSO, Nocodazole treatment, wild type 4 dpf Blebbistatin treatment.

We next explored whether cytoskeletal modulation also affects mechanical properties of the brain and in turn macrophage infiltration. We modulated the cytoskeleton by targeting tubulin expression using two pharmacological inhibitors, Nocodazole and Blebbistatin; Nocodazole inhibits the self‐assembly of tubulin and/or associated proteins. In vivo, it also depolymerizes preformed microtubules. Additionally, recent studies have demonstrated that Nocodazole treatment helped to differentiate the contributions of microtubules and actomyosin in regulating nuclear mechanics, chromatin accessibility, and early cellular responses to mechanical cues.^[^ ^47^, ^48^ ^]^ Brillouin frequency shifts decreased ≈150–400 MHz for all regions of the brain parenchyma compared to control (**Figure** **5**a,c). Nocodazole treatment led to the most significant decrease in BM shift and acetylated tubulin in the midbrain compared to the fore‐ and hindbrain (Figure 5b,d). Myosin II is known to influence cell contractility, which in turn can regulate tissue architecture. Additionally, Myosin II activity has been implicated in spindle orientation during epiboly, a crucial developmental stage in zebrafish.^[^ ^49^ ^]^ Thus, we also probed the role of Myosin II in regulating the mechanical properties of the brain parenchyma using the pharmacological agent, Blebbistatin (Figure 5a,c). Blebbistatin treatment altered brain tissue, evidenced by decreased normalized fluorescence intensity in acetylated tubulin staining (Figure 5b,d). Inhibition of Myosin II activity resulted in reduced Brillouin frequency shifts (15–50 MHz) across all brain parenchyma regions compared to control (Figure 5a,c). The regional stiffness trend persisted, with the hindbrain remaining the stiffest. However, the forebrain showed the most significant decrease in stiffness, with a 50 MHz Brillouin shift, and its acetylated tubulin intensity was most affected compared to other regions (Figure 5b,d). Given observed differences in macrophage infiltration related to development and mutant status, we investigated the effect of inhibiting mechanical properties through tubulin modulation. These treatments led to concomitant differences in the number of macrophages present in the midbrain where a reduction of infiltrated macrophages were detected following depolymerization of the tubulin and actin cytoskeleton (Figure 5e,f).

## 3 Discussion

This study sought to quantify tissue material properties across a wide frequency range. Our goal was to investigate if multimodal, scattering‐based approaches could elucidate changes affecting cellular decisions, even when measurements were taken at frequencies incongruent with known physiological timescales. In this respect, we explored whether a gigahertz‐scale material response could act as a proxy for understanding how tissue biophysical properties influence signaling events or behaviors that occur on much slower timescales. Moreover, we compared our measurements with a second optical‐based modality, Optical Trap based active microrheology. With these comparisons, we proceeded to investigate microglia‐mediated tissue remodeling. Microglia serve as the brain's primary innate immune defense in both zebrafish and mammals, playing a crucial role in the establishment and maintenance of neural components vital for cognitive and autonomic nervous system functions.^[^ ^6^, ^50^, ^51^ ^]^ In diseased states, the sentinel functions of these cells can be altered, with activated microglia potentially contributing to the progression of cancers and neurodegenerative diseases.^[^ ^6^, ^50^, ^51^, ^52^ ^]^ This study explored the bidirectional relationship between macrophages and tissue mechanical properties, specifically examining how tissue mechanics influence macrophage activation. Our research indicates that the brain's viscoelastic properties are significantly impacted by macrophages. This implies macrophages play an active role in preserving the brain's mechanical integrity, rather than simply reacting to injury. This research suggests that modulating macrophage activity or their interaction with the extracellular matrix could preserve tissue integrity by targeting mechanical changes. Restoring macrophage‐mediated tensional homeostasis through pharmacological, genetic, or biomaterial interventions may prevent or reverse pathological tissue remodeling. Ultimately, our work highlights the importance of considering the mechanical aspects of brain health, identifying macrophage‐mediated tensional homeostasis as a crucial regulatory and therapeutic target.

Scattering techniques, such as Raman spectroscopy, Optical Coherence Tomography, and Brillouin microscopy, are noninvasive.^[^ ^53^, ^54^, ^55^ ^]^ Brillouin microscopy assesses tissue mechanical properties by defining inelastic light scattering due to light interacting with acoustic phonons.^[^ ^55^ ^]^ Brillouin scattering occurs within nanoseconds to picoseconds, and these shifts, influenced by material properties, can be used as a proxy for tissue mechanical properties at the GHz scale. Brillouin microscopy faces penetration depth limitations due to sample turbidity. This issue has been quantitatively explored in several studies within the Brillouin field. In transparent tissues, penetration depth is virtually unlimited, with signal strength diminished only by optical aberrations.^[^ ^56^, ^57^ ^]^ Conversely, in turbid tissues, signal strength exhibits an expected exponential decay dependent on the sample's scattering coefficient.^[^ ^58^, ^59^, ^60^ ^]^ However, a key goal in using these techniques is establishing the mechanical signature obtained at the higher frequencies with additional mechanical mapping modalities. One such approach involves optical trap‐based active microrheology. Optical trapping‐based active microrheology applies forces to microscopic particles over durations ranging from milliseconds to seconds.^[^ ^61^ ^]^ This technique captures quasistatic or slow dynamic interactions, allowing for the inference of the material's underlying response.^[^ ^61^ ^]^ However, it requires well‐defined probes and careful calibration. Recent studies in the zebrafish show the broad utility of optical‐based techniques in probing biophysical properties, such as viscoelasticity and hemodynamic forces in vivo.^[^ ^22^, ^24^, ^62^, ^63^, ^64^, ^65^, ^66^, ^67^ ^]^ Here, we directly compare the two methods to provide a multifrequency analysis of microscale brain mechanics. Our data suggest that measured Brillouin shifts and OT‐AMR driven force deflections are largely driven by acetylated tubulin, microscale neuronal architecture, macrophage dynamics, and subcellular receptor modulation. We first show that there is good agreement between the techniques even though they probe mechanical properties at different timescales. BM probes the longitudinal modulus on the GHz timescale which differs from both the Young's and shear moduli measured by OT‐AMR. Importantly, the raw Brillouin shifts in line with the measurements obtained using the OT‐AMR correlated with the biological functions probed, such as mechanical heterogeneity as a function of brain region, development stage, and for multipronged perturbations of macrophage numbers and activity in vivo. These results further confirm congruence between Brillouin shifts and traditional rheological methods employed for cells and tissues.

However, there are some differences, such as the fact that a significant shift was not observed as the fish ages for BM measurements as observed for the OT‐AMR (Figure 1d,f; and Supporting Information 1f). This could be due to the contributions of the “viscous” component of the material that changes as a function of aging as we observed regional differences in viscous modulus as a function of age, where the forebrain became more “liquid‐like,” and the hindbrain became more “solid‐like” from 3 to 4 dpf. At lower frequencies, the brain tissue's mechanical properties are primarily influenced by its elastic component rather than its viscous component as seen from the OT‐AMR measurements (Figure 1c). BM probes GHz phonons and OT probes Hz–kHz particle fluctuations, both methods converge on the elastic‐dominated regime of tissue mechanics. Fractional viscoelastic models predict that storage moduli dominate in both the quasistatic and high‐frequency phonon regimes.^[^ ^68^ ^]^ This could be why BM (*M*′) shows a stronger correlation with OT *G*′ at these frequencies, as it is more sensitive to this elastic component (Figure 2e). This result falls in line with the comparison of BM with AFM measurements performed earlier on excised mammalian eyes, despite the differences in length scale.^[^ ^39^ ^]^ In addition, the power law analysis revealed age‐dependent transitions between semiflexible and flexible polymers at high frequencies (Supporting Information 1c).

We employed log–log analysis of data obtained from the two modalities to probe potential power‐law relationships across multiple decades of frequency and modulus values following similar approaches used for polymeric and biological viscoelastic materials. Simply can an Empirical relationship be gleaned from the multifrequency approaches (Figures 2e and 3h; and Supporting Information 2a,b, Supporting Information 4a,b). Converting Brillouin shifts to a modulus is an active area of research as the calculated modulus relies on both the density and local refractive index of the material. Understanding this conversion has recently become a greater focus due to its potential to introduce significant errors when mechanically interpreting data.^[^ ^40^ ^]^ Here, we estimated density by quantifying the number of cells per unit area in the measured regions. As for refractive index, its direct measurement is challenging in living animals. However, three main approaches have been employed to determine the refractive index (RI) of a sample as needed to convert Brillouin shifts into a Modulus. One approach assumes a homogeneous RI distribution, simplifying calculations but potentially leading to inaccuracies in nonuniform materials.^[^ ^39^, ^69^, ^70^ ^]^ The second approach posits a compensatory relationship between RI and absolute density, acknowledging the interplay between optical and physical properties.^[^ ^56^, ^57^, ^71^ ^]^ The third involves obtaining RI values from separate imaging setups, useful when direct in situ determination is difficult.^[^ ^40^, ^66^ ^]^ Here, we provided both an analysis based on the raw shift measurements and an analysis with a density correction, while keeping the refractive index constant. Not surprisingly, many of the raw correlation fits do not exhibit strong linearity in log–log analysis without considering the density factor. These only improved moderately when corrected using calculated region‐specific density values. While this simplification is an approximation, it allowed us to isolate the dominant effect of density variations on Brillouin modulus corrections for applying to developing brain tissue mechanics. In our hydrogels where we controlled for the concentration of the cells in a given 3D volume, we determined that the density factor correction now demonstrated that the hydrogel showed an increase in modulus as a function of cell numbers. Thus, our data may suggest that there are some conditions where density correction is critically needed to track apparent changes in moduli. However, cytoskeletal elements such as microtubules change both stoichiometry and organization as a function of development in hybrid tissues. These biological determinants will drive differences in the refractive indices and densities that are needed to interpret the longitudinal modulus as measured by BM (Figure 2b–f; and Supporting Information 2a,b). Thus, differences in both density, and refractive index, likely contribute to the imperfect correlations. We note that this is an important contribution and will be needed to fully understand the material response. Altogether, our measurements directly comparing these two modalities where the trends of stiffness measured using OT‐AMR agree well with the BS underscore that material properties even when measured at physiological timescales that do not yet correspond to known cellular processes are important proxies to assess mechano‐regulation noninvasively.

Here, these measurements provided insights into how tissue heterogeneity might influence macrophage dynamics and function. It has been previously shown that microglia migration is influenced by stiffness gradients within the brain parenchyma, a process known as durotaxis. Here, our data suggests that regional differences in the moduli of tissue can exert a significant impact on microglial behavior, affecting their ability to survey the brain, respond to injury, or contribute to neuroinflammation. Here, our in vitro assays employed stiffness assays to represent the extreme ends of the stiffness spectrum, compliant, and rigid, respectively. This then established controlled mechanical boundary conditions for macrophage mechanophenotyping. This approach combined with use of hydrogels whose rheological profiles matched the physical properties of brain extracellular matrices serve as reference points for assessing how macrophages perceive and adapt to their mechanical microenvironment.^[^ ^22^ ^]^ Our observations confirm that macrophages exhibit stiffness‐dependent phenotypes, even in simplified in vitro settings. Instead, they provide additional context for understanding how macrophage mechanosensing might function when tissue stiffness fluctuates outside of physiological ranges (≈0.1–1 kPa). These data, combined with our in vivo frequency‐resolved Brillouin and OT‐AMR measurements, more directly investigated the physiological relevance of viscoelastic dependence by probing the intrinsic mechanical spectrum of the developing brain. Collectively, these findings support a model where macrophages also react to stiffness cues in their morphology and gene expression. Moreover, we identified a specific set of macrophages, Csf1r+ macrophages as a key factor in brain mechanical integrity. We also determined that the brain mechanical properties are dependent on both the structural presence of these macrophages and the receptor that facilitates stromal remodeling. The mechanical homeostasis was modulated by both acute and permanent alterations of macrophage dynamics. Csf1r has pleiotropic functions in humans, rodents, and zebrafish where loss of the receptor resulted in central nervous system and skeletal deformities.^[^ ^72^ ^]^ In humans, variants contribute to a spectrum of development disorders that are classified collectively as BANDDOS: brain abnormalities, neurodegeneration, and dysosteosclerosis.^[^ ^73^ ^]^ More recently, variants of CSF1R have been implicated in adult‐onset leukodystrophy.^[^ ^74^ ^]^ The appearance of white matter lesions, axonal spheroids, and cerebral calcifications have deleterious effects on motor and cognitive functional resulting in death.^[^ ^74^ ^]^ Although our results are obtained using larval fish, these observations may have consequences for adult tissues. An important area for future research exists in bridging the knowledge gap between our microscale techniques and the macroscale techniques, such as magnetic resonance elastography which are already employed in the clinic.

Macrophages, both resident and recruited, play a dual role in cancer progression, either promoting or suppressing tumor growth, aiding tumor cell escape, and facilitating metastasis.^[^ ^75^, ^76^ ^]^ This has led to the exploration of macrophage‐specific receptors as therapeutic targets in cancer treatment.^[^ ^75^, ^76^, ^77^ ^]^ However, clinical trials have shown mixed results.^[^ ^78^ ^]^ A potential contributing factor to these inconsistent outcomes may be that targeting macrophage biochemical signaling could unintentionally alter the mechanical properties of the microenvironment. Here, we show that pharmacological modulation of csfr1 receptor significantly softens the brain in all regions. This treatment‐induced softening was even more pronounced than the removal of macrophages using small molecules and genetic ablation. Such alterations might either enhance or hinder the desired effects of tumor clearance in clinical settings. Therefore, a deeper understanding of how macrophage remodeling contributes to maintaining mechanical homeostasis is crucial for optimizing macrophage‐based therapies in disease management. Furthermore, it highlights the necessity of comprehending normal tissue homeostasis to effectively design microenvironment‐targeting therapeutics. Recently, a feedback loop involving pFAK and myosin IIa to regulate nuclear tension was shown to regulate chromatin accessibility via YAP–SWI/SNF complex remodeling.^[^ ^79^, ^80^ ^]^ Extrinsic tissue mechanical cues may activate intracellular pFAK–myosin II signaling and nuclear actin‐dependent transcriptional pathways. Here, we observed that macrophages primarily modified the ambient viscoelastic tissue milieu. Elucidating a comparable signaling cascade might provide additional insight to understand how macrophages preserve mechanical integrity.

## 4 Experimental Section

### Zebrafish Husbandry

Zebrafish were maintained at 28.5 °C on a 14‐h light/10‐h dark cycle according to standard procedures. Larvae were obtained from natural spawning, raised at 28.5 °C, and maintained in fish water, 60 mg sea salt (Instant Ocean, #SS15‐10) per liter of DI water. For all experiments, larvae were transferred to fish water supplemented with *N*‐phenylthiourea (PTU; Millipore Sigma, # P7629‐25G) between 18 and 22 h postfertilization to inhibit melanin formation for enhanced optical transparency. PTU fish embryo water was prepared by dissolving 400 µL of PTU stock (7.5% w/v in DMSO) per 1 L of fish water. Water was replaced twice per day.

### Zebrafish Lines

For wild type, AB* strain zebrafish were crossed for brain mechanics measurement at 3 and 4 dpf. Additionally, *csf1ra* mutant zebrafish (*panther* ^j4as^) were crossed to validate the role of *csf1ra* on brain mechanics and development at 3 and 4 dpf. To investigate the role of macrophage on brain mechanics, Tg(*mpeg1.1:NTR‐IRES‐eGFP‐CAAX*)^co57^ were utilized where the macrophage specific *mpeg1.1* promoter, drives expression of the *e. coli* Ntr and eGFP. Tg(*fli1a:eGFP*)^y1^ zebrafish were used to visualize the integrity of blood–brain barrier upon 150 kDa dextran circulation injection at 3 and 4 dpf compared to *panther* fish. For all experiments, at around 22–24 hpf, larvae were transferred to fish water supplemented with PTU to inhibit melanin formation for increased optical accessibility. Larvae were maintained in an incubator at 28.5 °C and checked for normal development.

### Optical Tweezer Based Active Microrheology (OT‐AMR) Measurement

Zebrafish larvae at 2 dpf were anesthetized using 0.4% buffered tricaine methanesulfonate (Syndel SYNCAINE Cat. No. TRS1). 2 nL of 1 µm polystyrene beads (ThermoFisher Scientific, #F8816) resuspended in Phosphate Buffered Saline at a final concentration of 10^7^ beads mL^−1^ was injected directly into brain parenchyma (midbrain). Injected larvae were transferred into fresh PTU water and maintained at 28.5 °C. For OT‐AMR measurement, injected larvae at 3 and 4 dpf were anesthetized using 0.4% buffered tricaine and embedded in 1.25% low melting point agarose gel and allowed to polymerize in with cover glass (no. 1.5 thickness). Then, fish water supplemented with tricaine was added to the agarose hydrogel for the entire time of data acquisition as previously described.^[^ ^65^, ^81^ ^]^ The details of the home‐built OT‐AMR setup are described.^[^ ^22^, ^24^ ^]^ Mainly, the equipment is composed of two near infrared lasers, a 1064 nm trapping laser (IPG Photonics, #YLR 20 1064 Y11) to trap and oscillate a bead and a 975 nm detection laser (Lumics, #LU0975M00 1002F10D) to detect the movement of bead. Along with the two near infrared lasers, there are two quadrant photodiodes (QPD; First Sensor, #QP154‐QHVSD) to detect trapping laser displacement (trap QPD) and the motion of bead (detection QPD). The trap laser is oscillated by a dual axis acousto‐optic deflector (AOD; IntraAction, #DTD274HD6), which is operated by radio frequency generating cards (Analog Devices, #AD9854/PCBZ) with on‐board temperature‐controlled crystal oscillators.^[^ ^22^, ^24^ ^]^ Then, the cards are digitally controlled by a data acquisition (DAQ) card (National Instruments, PCIe 5871R FPGA). Right after AOD, a small fraction of trap laser power (≈1%) is directed to trap QPD. Both trapping and detection lasers go through the backport of an inverted microscope (Nikon, Eclipse Ti‐U) with a long working distance water immersive objective (Nikon, MRDO7602 CFIPLAN APO VC60XA WI 1.2 NA). Then, a long working distance (WD) and high numerical aperture (NA) condenser (Nikon, WI 0.9NA) collects the light from objective. After the condenser, a dichroic mirror (Chroma, ZT1064RDC‐2P) reflects both trap and detection lasers. A bandpass filter (Chroma, #ET980/20x) excludes the trap laser so that the detection laser reaches detection QPD to record the motion of bead. Then, time‐correlated trap and detection QPD signals from control and samples are collected and conducted by the DAQ card and custom program LabVIEW (National Instruments). During experiments, condenser is placed in Kohler illumination to locate beads via a piezo XYZ nanopositioning stage (Prior, #77 011 201) and a charge‐coupled device (CCD) camera (Andor, Ixon DU‐987E‐C50‐#BV). A selected bead is precisely positioned at the center of trap after scanning it by detection laser in three dimensions using a piezo nanopositioning while recording the voltages (V) from the detection QPD. The relationship between V and displacement (nm) relationship, β, from the detection QPD is calibrated in situ by fitting the central linear line of the detector responses to scanning the bead through the detection laser in the direction of the trap laser oscillations, yielding β in V/nm. A trap QPD records the position of the trap laser to find the relative phase lag between the trap oscillations and bead. The trap stiffness, *k*, is determined in situ for selected beads based on the active‐passive calibration method.^[^ ^24^ ^]^ As described by Fischer and Berg‐Sørensen, the stiffness, *k*, is determined based on the active power spectrum, ${\tilde{R}}_{L}\left(\omega \right)$, and passive power spectrum, *P*_U_(ω).^[^ ^82^ ^]^ The stiffness (Equation (1)) is

(1) $$k=\frac{ℜ\left\{{\tilde{R}}_{L}\left(\omega \right)\right\}}{P_{U}\left(\omega \right)}$$

Active power spectrum, ${\tilde{R}}_{L}\left(\omega \right)$, is recorded while trap laser is oscillating. The active power spectrum (Equation (2)) is

(2) $${\tilde{R}}_{L}\left(\omega \right)\equiv \frac{{\tilde{x}}_{\mathrm{dr}}\left(\omega \right)}{-i\omega {\tilde{x}}_{L}\left(\omega \right)}$$

where ${\tilde{x}}_{L}\left(\omega \right)$ and ${\tilde{x}}_{\mathrm{dr}}\left(\omega \right)$ are the Fourier transforms of the time series of the positions of the trap laser and the driven bead, respectively.

Passive power spectrum, *P*_U_(ω), is measured while trap laser is held stationary. The passive power spectrum (Equation (3)) is

(3) $$P_{U}\left(\omega \right)\equiv \langle {\left|{\tilde{x}}_{U}\left(\omega \right)\right|}^{2}\rangle$$

where ${\tilde{x}}_{U}\left(\omega \right)$ is the Fourier transform of the time series of the undriven bead's thermally fluctuating position while trap laser is stationary.

With all the information from the trajectories of bead positions along with β, *k*, mass of bead (*m*), and radius (*a*), the generalized Stokes–Einstein relationship yields the complex modulus, G*(ω), shown in (Equation (4))

(4) $$G^{*}\left(\omega \right)=\frac{i\omega {\tilde{\gamma }}_{D}\left(\omega \right)}{6\pi a}$$

where ${\tilde{\gamma }}_{D}\left(\omega \right)$ corresponds to the friction relaxation spectrum (Equation (5)) based on the active power spectrum, ${\tilde{R}}_{L}\left(\omega \right)$

(5) $${\tilde{\gamma }}_{D}\left(\omega \right)+i\omega m=-\frac{k}{i\omega }\left(\frac{1}{i\omega {\tilde{R}}_{L}\left(\omega \right)}+1\right)$$

Then, the complex modulus can be broken into elastic modulus, *G*′(ω), and viscous modulus, *G*′′(ω) (Equation (6))

(6)

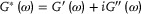




For all the measurements, laser power was set to 100 mW at the microscope back port, while the amplitude of trap laser oscillations was set to 100 nm. Experiments were controlled using custom LabVIEW programs.

### Brillouin Microscopy

Larvae were anesthetized using 0.4% buffered tricaine and embedded in a dorsal orientation in 1.25% low melting point agarose gel and allowed to polymerize in with cover glass (no. 1.5 thickness) at 3 and 4 dpf. Dorsal orientation has been chosen to scan all brain regions (forebrain, midbrain, and hindbrain) but also to avoid eye interference during data acquisition. Then, fish water supplemented with tricaine was added to the agarose hydrogel for the entire time of measurement. Brillouin microscopy setup has been described elsewhere.^[^ ^83^, ^84^ ^]^ Briefly, the setup is composed of 660 nm laser (Laser Quantum, #Torus‐660), which illuminates the larvae brain at 20–30 mW via 40x air objective (Olympus, LUCPLFLN40X 0.6 NA) after passing through the backport of microscopy body (Olympus, #IX81+DSU). Then, the backscattering from the focused voxel is collected through a single mode fiber (Thorlabs, #P1‐460Y‐FC‐2), which serves as confocal pinhole. Brillouin light is then directed and analyzed by a two‐stage VIPA etalon (FSR 15 GHz, LightMachinery, #OP‐6721–6743‐3) spectrometer in cross‐axis configuration.^[^ ^85^ ^]^ The VIPA spectrometer separates the frequencies of light, which is imaged on a high‐sensitivity electron‐multiplying charge‐coupled device (EMCCD) camera (Andor, #iXon 897). Adjustable slits in the spectrometer blocks the stray light from elastic scattering to yield two Brillouin peaks, which are the anti‐Stokes Brillouin scattering peak and the Stokes peak of the next diffraction order. At the end, the graph of intensity versus two Brillouin peaks (frequency) is obtained. The raw data were fitted with a Lorentzian function in a custom MATLAB program based on nonlinear least squares fitting to localize peak centers between the two Brillouin peaks. Brillouin microscopy measures spontaneous Brillouin scattering from the interactions between the incident light and inherent acoustic phonons (thermal fluctuations) inside the sample. Then, a frequency shift or Brillouin shift can be measured from the scattering. The Brillouin shift, *v*_B_, is calculated based on (Equation (7))

(7) $$v_{B}=\frac{2n}{\lambda }\sqrt{\frac{M^{\prime }}{\rho }}\sin\left(\frac{\theta }{2}\right)$$

where 𝑛 is refractive index of the material, 𝜆 is the laser wavelength, 𝑀′ is the longitudinal elastic modulus of measured sample, 𝜌 is the density, and 𝜃 is the collection angle of the scattered light. For the setup, backward scattered light was collected, yielding 𝜃 = 180°. Before actual sample measurements, Brillouin scattering calibration has been done by collecting 500 Brillouin spectra of methanol and water at 10 ms exposure time. With the known literature values of Brillouin shift for methanol and water, the effective free spectral range and the spectral dispersion parameter (GHz per pixel) could be calculated. Calibration was done at least once an hour throughout whole measurements. The experimental parameters for brain scanning were done at 50–100 ms exposure time and a pixel size at 2 µm × 2 µm.

Corrected Brillouin modulus was calculated based on the conversion of experimental Brillouin shifts from each brain regions to longitudinal modulus (𝑀′). The longitudinal modulus is correlated to OT‐AMR's complex modulus (lG*l), elastic modulus (*G*′), and viscous modulus (*G*″) at 7 Hz, 907 Hz, and 15 kHz based on log‐log linear relationship, log(𝑀′) = a* log(lG*l, *G*′, or *G*″) + b, where the relative change in Brillouin modulus is related to the relative change in OT‐AMR *G*′. Thus, the slope (a) is multiplied by log(*G*′) to get the corrected Brillouin modulus, where log(Brillouin modulus) = a * log(*G*′) + log(*M*′). During the conversion process, the values of 1.04 g mL^−1^ density and the refractive index of 1.35 from a HeLa cell^[^ ^40^, ^41^ ^]^ are kept constant for raw correlation analysis between OT‐AMR and Brillouin microscopy to understand the role of density factor correction.

### Drug Treatments

‐ *Myosin II inhibition*: (±)‐Blebbistatin (Millipore Sigma, #203390‐5MG) was diluted to 10 µm in 0.1% DMSO in PTU fish embryo water right before use to inhibit nonmuscle Myosin II. Larvae were treated overnight (≈16 h) from 3 dpf. The control was 0.1% DMSO in PTU fish embryo water.

‐ *Acetylated tubulin perturbation*: Nocodazole (Selleckchem, #S2775) was diluted to 10 µm in 0.1% DMSO in PTU fish embryo water right before use to decrease acetylated tubulin. Larvae were treated overnight (≈16 h) from 3 dpf. The control was 0.1% DMSO in PTU fish embryo water.

‐ *Macrophage ablation*: Metronidazole (Millipore Sigma, #M1547) was diluted to 10 mm in 0.1% DMSO in embryo medium immediately before use for nitroreductase‐mediated cell ablation. The control was 0.1% DMSO in PTU fish embryo water. As Metronidazole is light sensitive, all treatment groups were kept in the incubator in the dark including control groups. The treatment started at 3 dpf for overnight (≈16 h).

‐*Macrophage depletion injection*: Zebrafish larvae at 3 dpf were anesthetized using 0.4% buffered tricaine. 1.2 nL of clodronate liposomes (FormuMax, #F7010C‐AH) was injected directly into brain parenchyma (midbrain) to deplete macrophages in the brain. As a control, 1.2 nL of empty liposomes (FormuMax, #F70101‐AH) was injected. Injected larvae were immediately transferred into fresh PTU fish embryo water.

‐*CSF1R inhibitor*: PLX5622 (Selleckchem, #S8874‐5 mg) was diluted to 10 µm in 0.1% DMSO in PTU fish embryo water right before use to inhibit CSF1R. Larvae were treated overnight (≈16 h) from 3 dpf. The control was 0.1% DMSO in PTU fish embryo water.

### Macrophage Labeling

Neutral red (Millipore Sigma, # N7005) was diluted to 2.5 µg mL^−1^ in embryo medium and administered to 3‐4 dpf larvae for 2.5 h at 28.5 °C in the dark. Following staining, larvae were washed two or three times in fresh PTU fish embryo water and monitored until nonspecific tissue redness had washed out (30–60 min), before mounting dorsal‐down in low‐melt agarose for brightfield imaging of optic tectum (midbrain) microglia.

### Zebrafish Immunofluorescence and Approximate Brain Density Calculation

Larvae from 3 and 4dpf were fixed for 4 h at 4 °C in 4% paraformaldehyde in PBS. Fixed larvae were washed three times in PBDT (PBS supplemented with 1% DMSO and 0.5% Triton X‐100). Washed larvae were gone sequential dehydration to methanol and then were stored at −20 °C for at least 12 °C h. Dehydrated larvae in methanol were sequentially hydrated in PBDT. Then, the larvae were permeabilized with 10 µg mL^−1^ Proteinase K (Millipore Sigma, #3 115 879 001) in PBDT for 15 min at room temperature to remove the epidermis, followed by fixation in 4% paraformaldehyde in PBS for 30 min. Larvae were washed three times with PBDT and blocked for 1 h at room temperature in PBDT containing 5% goat serum. Larvae were then incubated in a 1:200 dilution of mouse monoclonal antitubulin (acetal Lys40) antibody (GeneTex, #16 292) in PBDT with 5% goat serum for at least 3 days at 4 °C to stain acetylated tubulin. Stained larvae were washed quickly in PBDT three times and then washed an additional two times in PBDT for 15 min each. Antibody‐stained larvae were then transferred to a 1:250 secondary antibody containing an AlexaFluor Plus 488 goat antimouse secondary antibody (Thermo Fisher Scientific, #A‐11001) in PBDT with 5% goat serum. The larvae were stained for overnight at 4 °C. Then, the fish were washed three times in PBDT for 15 min each and imaged with a Zeiss 780 LSM confocal microscopy.

Confocal z‐stacks were acquired at 0.5 µm steps with the pinhole diameter set at 90.1 µm. 12‐bit images were acquired with a Zeiss 20x EC Plan‐Apochromat, 0.8 NA objective. Samples were excited with 488 nm light from an argon laser at 2% total power of 25 mW for normalized fluorescence intensity comparison. Transmittance spectrum was also recorded. The master gain was set at or below 650. Pixel dwell times of 1.58 ms were used. Then, images were max projected in terms of the *z*‐axis and the *y*‐axis based on Omer et al. XYZ projection tool^[^ ^86^ ^]^ for further image analysis by using ImageJ. Each brain region was quantified based on acetylated tubulin area to get volume change in terms of xz and xy area, based on Marchant et al. acetylated tubulin immunofluorescence normalization protocol.^[^ ^36^ ^]^ The brain tissue was dissociated into single cells using Bresciani et al. protocol for dissociation with 20 µL Collagenase 100 mg mL^−1^ per 480 µL 0.25% trypsin‐EDTA per 15 zebrafish to get estimated brain tissue mass.^[^ ^87^ ^]^

### Ex Vivo and In Vitro of Hyaluronic Acid Gel Mechanical Remodeling by Macrophages

Brain tissue (from fish that were more than 1 year old) was dissociated into single cells using the protocol for dissociation as described in Bresciani et al.^[^ ^87^ ^]^ Briefly, a mixture of 40 µL^−1^ Collagenase 100 mg mL^−1^ per 460 µL^−1^ 0.25% trypsin‐EDTA was added for every adult zebrafish. Following disassociation, Mpeg1.1:GFP+. Positive cells (top 2% based on GFP intensity) were isolated via Fluorescence‐Activated Cell Sorting (FACS) into PBS. Expression of GFP was confirmed by fluorescence microscopy. Mouse microglia, EOC2 (ATCC, CRL‐2467), were cultured in 70% Dulbecco's Modified Eagle's Medium (Gibco Cat no. 10313‐021) with 4 mm L‐glutamine adjusted to contain 1.5 g L^−1^ sodium bicarbonate and 4.5 g L^−1^ glucose; 10% fetal bovine serum; 20% LADMAC conditioned media (produced from the LADMAC cell line (ATCC CRL‐2420) grown in Eagle's Minimum Essential Medium with 10% fetal bovine serum for 2 days).

Zebrafish macrophages (pre‐sorted) or cultured mouse microglia (EOC2) were then resuspended in growth medium at concentrations of 1 × 10^6^ or 5 × 10^6^ cells mL^−1^. A cell‐ ECM mixture was prepared by combining 40 µL of this cell solution with 200 µL of hyaluronic acid gel (Advanced Biomatrix, #GS310F). For each well, 240 µL of the cell/matrix mixture was plated in glass‐bottom dishes and incubated for 1 h at 37 °C (or 28.5 °C for zebrafish). Following matrix gelation, media (with or without pharmacological agents) was introduced. The cell/matrix was then subjected to mechanical imaging using Brillouin microscopy during 24‐ to 28‐h measurement windows. The density factor is critical for interpreting Brillouin microscopy data because the Brillouin shift is inversely proportional to the square root of the material density; thus, higher density results in a lower Brillouin shift. In the experiments, the sample consisted of 200 µL of hyaluronic acid (HA) and 40 µL of media containing cells. Therefore, variations in cell concentration contribute only partially to the overall density change. Comparing two conditions with cell densities of 5 million cells per milliliter versus 1 million cells per milliliter represents a fivefold increase in cell mass, assuming the 40 µL volume remains constant. Consequently, the maximum relative density correction factor affecting the Brillouin shift or longitudinal modulus (*M*′) can be estimated as (1/6) × 5 = 5/6, which accounts for the contribution of cell density within the mixture. Taking the square root of this factor, (5/6)^0.5 ≈ 0.912, indicates that relative *M*′ should be corrected by the reciprocal, ≈1/0.912 = 1.09.

### High‐Throughput Zebrafish Imaging via Vertebrate Automated Screening Technology (VAST)

At 2 dpf, embryos were put into a flat‐bottom 96‐well plate in 150 µL fish water containing PTU. Using LP Sampler (Union Biometrica), fish were loaded into a capillary and imaged by a Vertebrate Automated Screening Technology (VAST) BioImager (Union Biometrica). The camera of the VAST BioImager was used to quantify brain development and morphology. The fish were scored based on the development of the hindbrain. After acquisition, the embryos were dispensed into a 96‐well plate, rinsed from tricaine, and placed back into the incubator.

### RNA Isolation, Bulk RNA Sequencing, and Quantitative Real‐Time PCR

RNA from embryos was extracted using TriZol (Thermo Fisher Scientific Cat No 15 596 026) according to the manufacturer protocol. 1.25 µg of Total RNA was submitted for RNA‐sequencing to the NCI CCR Genomics Core. The libraries were made using the Poly(A) Selection kit (Illumina). 150 bp fragments were sequenced by paired‐end 75 bp utilizing Illumina NextSeq. Reads were aligned to Ensembl Zebrafish Genome Build 11, GRCz11, using Hisat2. SAMtools was used for sorting and indexing. PartekFlow was used for ANOVA and pathway analysis.

RNA from microglia cells was extracted using TriZol according to the manufacturer protocol. 1 µg of total RNA was used to make cDNA using oligod(T)_16′_ and TaqMan Reverse Transcription Reagents (Applied Biosystems #N8080234) in a 20‐µL reaction using the manufacturer protocol. TaqMan assays were run using 1 µm of the cDNA reaction in a duplex quantitative polymerase chain reaction using TaqMan Gene Expression Master Mix (Applied Biosystems #4 369 016) according to the manufacturer's protocol. Gene expression was normalized using a VIC‐labeled probe for Rn18s;Rn4+ (Mm01257265_m1). Predesigned and FAM‐labeled TaqMan probes from Applied Biosystems were used for Csf1r (Mm01266652_m1) and Tnf (Mm00443258_m1). The experiment involved three biological replicates, each with three technical replicates. For each biological replicate, cycle thresholds for every transcript of interest were normalized against a corresponding Rn18s;Rn4+ replicate across the three technical replicates. These technical replicates were then averaged to determine a mean ΔCT and a fold‐change (ΔΔCT), allowing for comparison between mouse microglia on a soft substrate (0.2 kPa). (Advanced Biomatrix, #5165‐5EA) and stiff substrate (glass) for ≈24 h. Results from the three biological replicates were then used to calculate the average fold‐changes and standard deviations.

### Statistical and Data Analysis

All statistical analyses were carried out using GraphPad Prism (v10.1). Additional data processing utilized platform‐specific software, including custom LabVIEW code for Optical Trap‐based Active Microrheology (OT‐AMR) control and custom MATLAB scripts for fitting raw OT‐AMR and Brillouin microscopy datasets. ImageJ was used for image analysis. PartekFlow facilitated ANOVA and pathway enrichment analyses of bulk RNA‐sequencing data.

*Statistical testing*: A paired two‐tailed *t*‐test with unequal variances was used to evaluate intra‐regional mechanical heterogeneity. Unpaired two‐tailed *t*‐tests with unequal variances were used to assess drug treatment effects. Statistical significance was defined as *p* < 0.05 (*), *p* < 0.01 (**), *p* < 0.001 (***), and *p* < 0.0001 (****). All tests were performed with a 95% confidence interval. Exact statistical parameters, including test type, *n*, and *p*‐values, are provided in the figure captions.

*Mechanical measurements (in vivo and ex vivo)*: Raw OT‐AMR and Brillouin data from three independent in vivo and in vitro experiments (hyaluronic acid hydrogels) were fitted with custom MATLAB scripts. Brillouin frequency shifts and complex moduli (|G*|) were normalized to the mean wild‐type hindbrain value at 3 days postfertilization (dpf). Log–log plots were employed for correlation analyses between *M*′ (Brillouin microscopy) and *G*′ (OT‐AMR). Density‐factor corrections were applied using the relationship log(*M*′) = a·log(|G*|, *G*′, or *G*″) + b.

*Imaging*: Quantitative analysis of acetylated tubulin immunofluorescence and in vivo neutral red staining was performed in ImageJ using data from three independent experiments. Acetylated tubulin intensity was normalized across developmental stage (3–4 dpf), csf1ra genotype (wild‐type vs null), and treatment condition (DMSO/control vs inhibitor). Neutral red–positive macrophages were quantified by counting phagocytic puncta. Only viable larvae (verified by normal heartbeat and development staging) were included, with a minimum of four embryos (fixed or live) analyzed per condition for each experiment.

*Transcriptomics*: Bulk RNA‐sequencing was conducted in two independent biological replicates at 2 and 4 dpf, comparing csf1raWT and csf1ranull larvae. ANOVA and pathway analyses were conducted in PartekFlow.

*qRT‐PCR*: Gene expression was normalized to the housekeeping gene Rn18s;Rn4+. Fold changes (ΔΔCT) were calculated across three independent experiments comparing soft versus stiff conditions.

*Data presentation*: For OT‐AMR measurements, data are presented as mean ± standard error of the mean (s.e.m.). All other datasets are reported as mean ± standard deviation (s.d.). All values represent independent biological replicates, with sample sizes (*n*) provided in the respective figure legends.

### Ethical Statement

All animal experiments were done under protocols, LCB‐ 029 and LCB‐031 approved by the National Cancer Institute (NCI) and the National Institutes of Health (NIH) Animal Care and Use Committee.

## Conflict of Interest

The authors declare no conflict of interest.

## Supporting information



Supporting Information

## Data Availability

The data that support the findings of this study are available from the corresponding author upon reasonable request.
