# Supplementary material for: Macrophages Mediate Mesoscale Brain Mechanical Homeostasis
Source: Adv Mater. 2025 Nov 18;38(6):e17493. doi: 10.1002/adma.202517493 (PMC12848650; doi:10.1002/adma.202517493)
Supplement: Supplementary file 1 — Supporting Information [file ADMA-38-e17493-s001.docx]

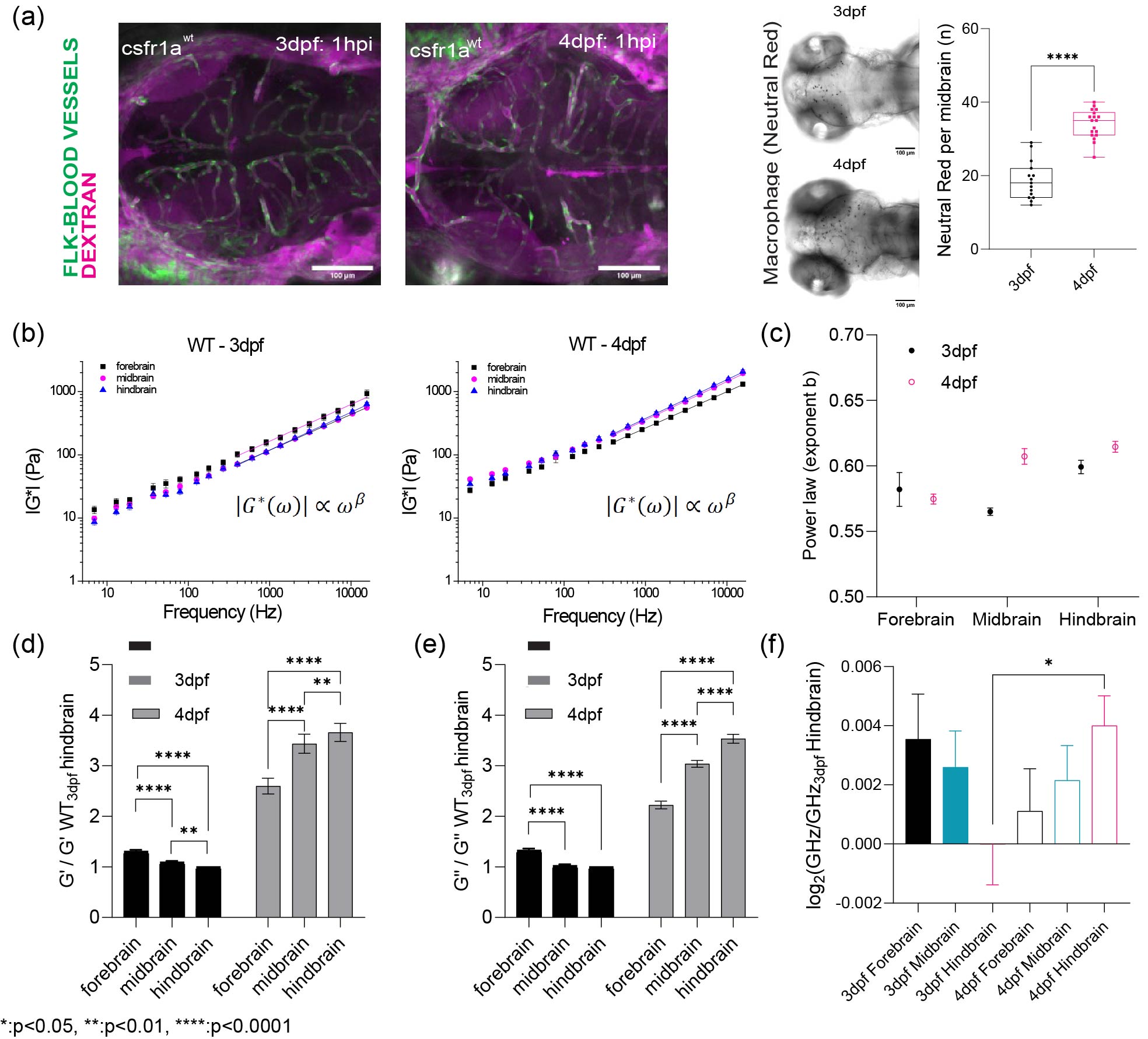


**Extended Data1:**

(a) Micrographs following injection of 150kDa Dextran into blood circulation to assess the integrity of blood-brain barrier for wild type during development. Micrographs showing macrophages in the midbrain labelled by neutral red assay for wild type larval zebrafish as a function of development (wild type 3 dpf: n = 15 and wild type 4 dpf: n = 18). **** p<0.0001, unpaired two-tailed t-tests; confidence interval = 95%. (b) Log-log plots showing the comparison of broadband values obtained using OT-AMR of 3 dpf wild-type (WT) brain mechanical property (|G*|) for the frequency range from 7 Hz to 15 kHz with frequency-dependent power law fit (from 400 Hz to 15 kHz) , as a function of brain regions (forebrain = black, midbrain = magenta, and hindbrain = cyan) (Left panel). Log-log plots showing the comparison of broadband values obtained using OT-AMR of 4 dpf wild-type (WT) brain mechanical property (|G*|) for the frequency range from 7 Hz to 15 kHz with frequency-dependent power law fit (from 400 Hz to 15 kHz), as a function of brain regions (forebrain = black, midbrain = magenta, and hindbrain = cyan) (Right panel). (c) the exponent β of power law for wild type during development and in different brain regions (d) a bar graph of normalized wild-type 3 and 4 dpf elastic modulus (G’) obtained using OT-AMR with respect to wild-type 3 dpf hindbrain averaged across all the frequencies. ** p<0.01, **** p<0.0001, paired two-tailed t-tests; confidence interval = 95%. (e) a bar graph of normalized wild-type 3 and 4 dpf viscous modulus (G’’) obtained using OT-AMR with respect to wild-type 3 dpf hindbrain averaged across all the frequencies. **** p<0.0001, paired two-tailed t-tests; confidence interval = 95% (f) Bar graph for log2 of normalized Brillouin shifts of wild-type 3 and 4 dpf with respect to the average Brillouin shift measured from the wild-type 3 dpf hindbrain. * p<0.05, unpaired two-tailed t-tests; confidence interval = 95%.

**
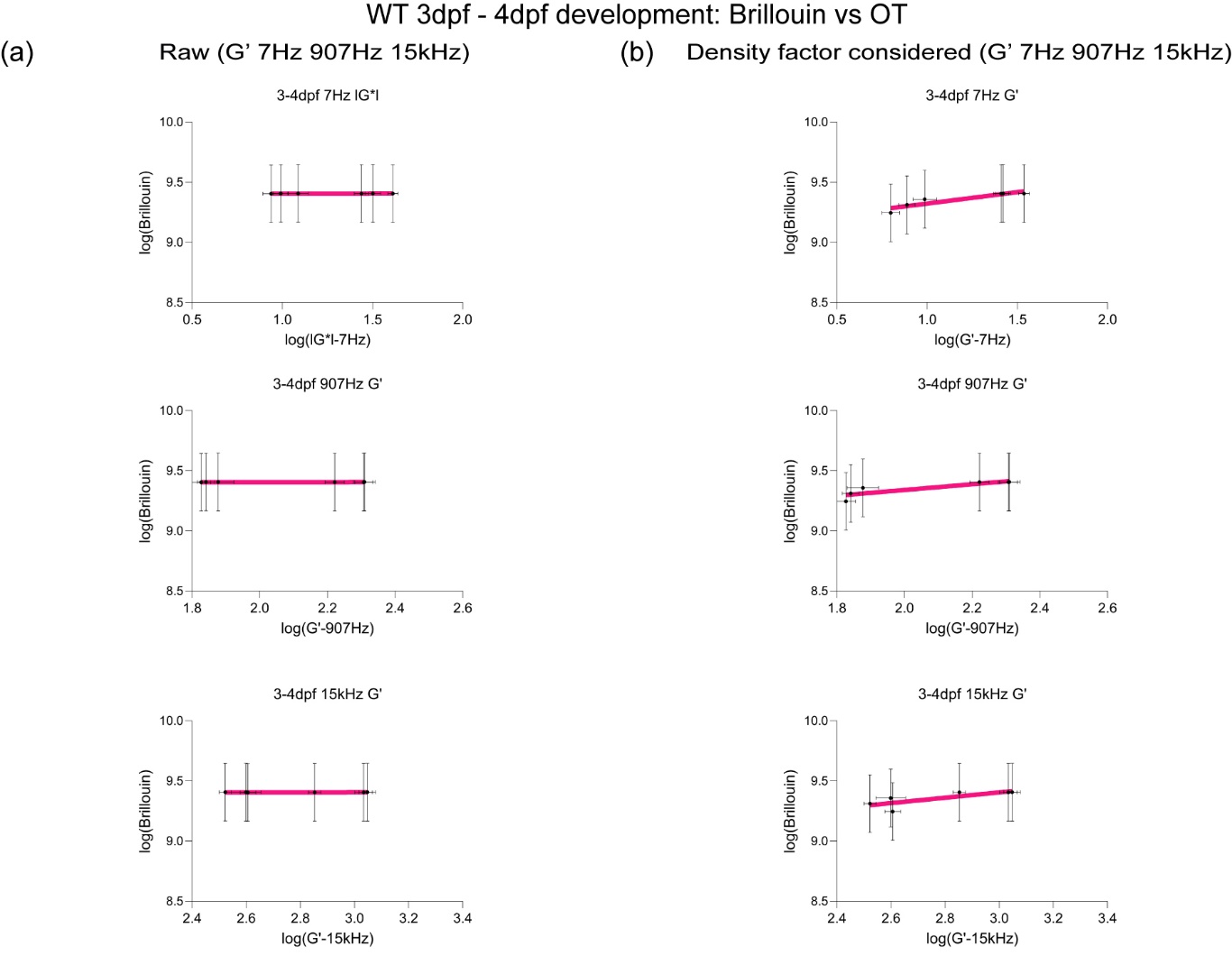
**

**Extended Data2:**

(a) Linear fit (top) between log(G’ for forebrain, midbrain, and hindbrain) and log(Brillouin modulus M’ calculated based on estimated brain refractive index and density) to get a slope to get corrected Brillouin modulus (bottom) in respect to G’ at 7 Hz, 907 Hz, and 15 kHz during development (3 to 4 dpf). (b) Linear fit (top) between log(G’ for forebrain, midbrain, and hindbrain) and log(Brillouin modulus M’ calculated based on estimated brain refractive index and density after density factor correction) to obtain corrected Brillouin modulus (bottom) in respect to G’ at 7 Hz, 907 Hz, and 15 kHz during development (3 to 4 dpf).


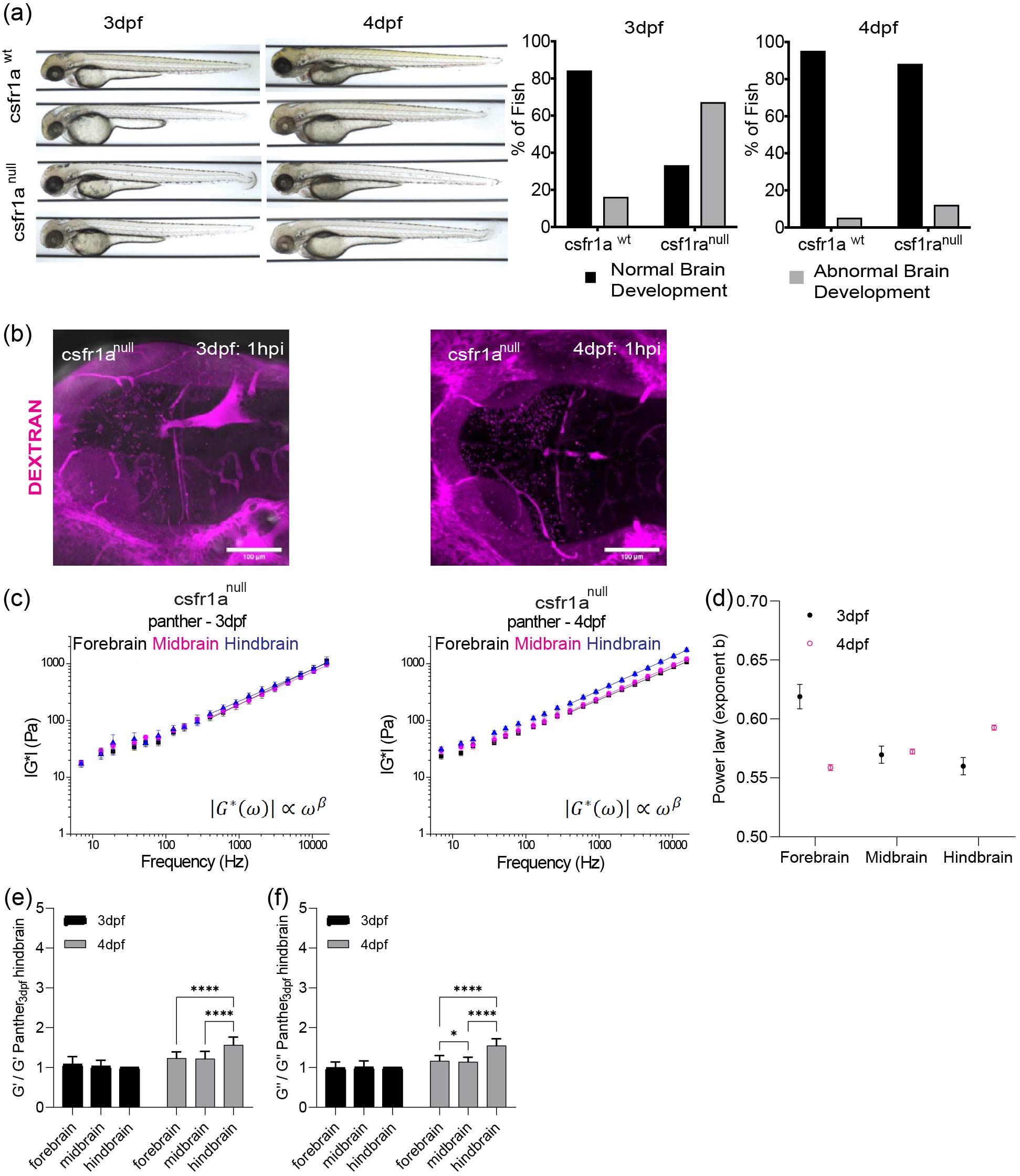


**Extended Data3:**(a) Micrographs of larval fish to assess the role of *csf1ra* on brain development at 3 dpf and 4 dpf *via* VAST imaging (left panel), graphs quantifying the distribution of normal and abnormal brain development as a function of age and mutant status ( right panel) (b) circulation injection of 150kDa Dextran to show the integrity of blood-brain barrier of the ^null^ mutant during development (c) Log-log plots showing the comparison of broadband values obtained using OT-AMR of 3 dpf c*sf1ra*^null^ brain mechanical property (|G*|) for the frequency range from 7 Hz to 15 kHz with frequency-dependent power law fit (from 400 Hz to 15 kHz), as a function of brain regions (forebrain = black, midbrain = magenta, and hindbrain = cyan) (Left panel). Log-log plots showing the comparison of broadband values obtained using OT-AMR of 4 dpf c*sf1ra*^null^ brain mechanical property (|G*|) for the frequency range from 7 Hz to 15 kHz with frequency-dependent power law fit (from 400 Hz to 15 kHz), as a function of brain regions (forebrain = black, midbrain = magenta, and hindbrain = cyan) (Right panel). (d) the exponent β of power law for c*sf1ra*^null^ during development and in different brain regions (e) a bar graph of normalized c*sf1ra*^null^ 3 and 4 dpf elastic modulus (G’) obtained using OT-AMR with respect to c*sf1ra*^null^ 3 dpf hindbrain averaged across all the frequencies. **** p<0.0001, paired two-tailed t-tests; confidence interval = 95%. (f) a bar graph of normalized c*sf1ra*^null^ 3 and 4 dpf viscous modulus (G’’) obtained using OT-AMR with respect to c*sf1ra*^null^ 3 dpf hindbrain averaged across all the frequencies. * p<0.05, **** p<0.0001, paired two-tailed t-tests; confidence interval = 95%.


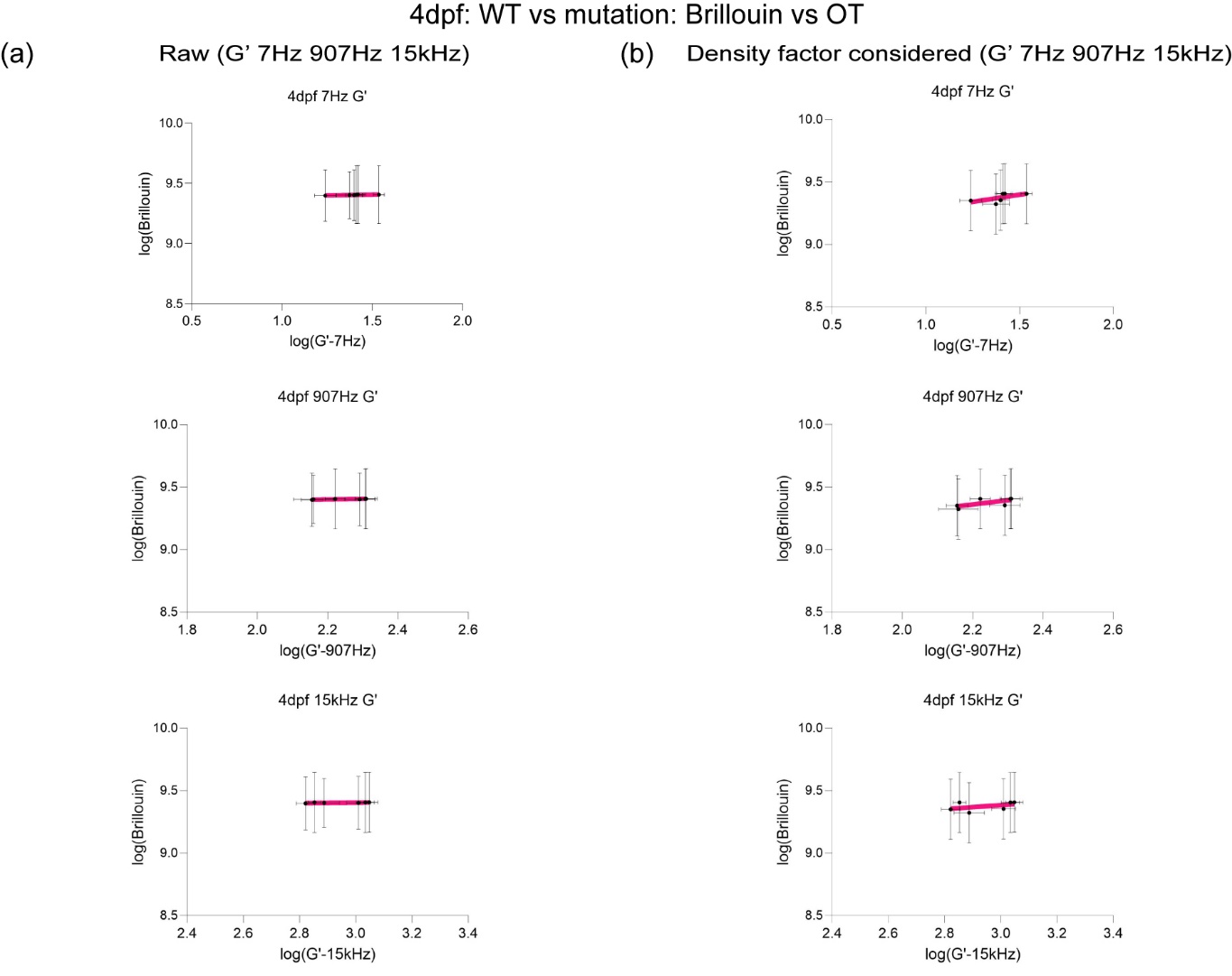


**Extended Data4:**

(a) Linear fit (top) between log(G’ for forebrain, midbrain, and hindbrain) and log(Brillouin modulus M’ calculated based on estimated brain refractive index and density) to obtain corrected Brillouin modulus (bottom) in respect to G’ at 7 Hz, 907 Hz, and 15 kHz for *csf1ra*^null^ mutation at 4 dpf. (b) Linear fit (top) between log(G’ for forebrain, midbrain, and hindbrain) and log(Brillouin modulus M’ calculated based on estimated brain refractive index and density after density factor correction) to get a slope to get corrected Brillouin modulus (bottom) in respect to G’ at 7 Hz, 907 Hz, and 15 kHz for *csf1ra*^null^ mutation at 4 dpf.

**
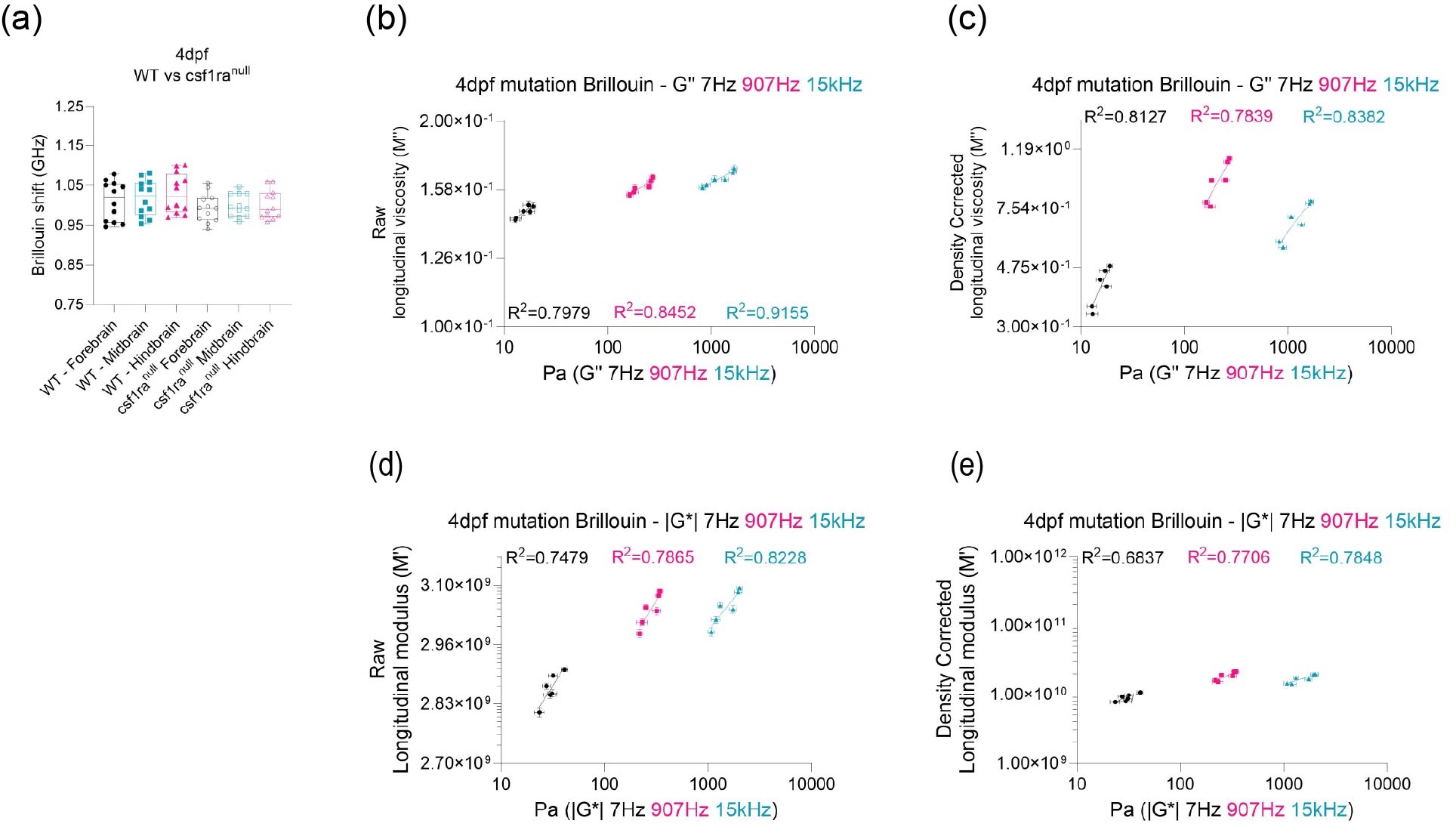
**

**Extended Data5:**

(a) Graphs depicting the average Brillouin shifts (line width) for each region (‘Fore-’, ‘Mid-’, and ‘Hind-’), comparing with wild type (wild type = 12 and *csf1ra*^null^ = 12). (b) Raw correlation between corrected Brillouin viscosity (M’’) and OT-AMR viscous modulus (G’’) at 7 Hz, 907 Hz, and 15 kHz at *csf1ra* mutational status at 4 dpf in log-log plot (c) Density factor corrected correlation between corrected Brillouin viscosity (M’’) and OT-AMR viscous modulus (G’’) at 7 Hz, 907 Hz, and 15 kHz at *csf1ra* mutational status at 4 dpf in log-log plot. (d) Raw correlation between corrected Brillouin (longitudinal) modulus (M’) and OT-AMR complex modulus (|G*|) at 7 Hz, 907 Hz, and 15 kHz for *csf1ra*^null^ mutant at 4 dpf in log-log plot. (e) Density factor corrected correlation between corrected Brillouin (longitudinal) modulus (M’) and OT-AMR complex modulus (|G*|) at 7 Hz, 907 Hz, and 15 kHz for *csf1ra*^null^ mutation at 4 dpf in log-log plot.


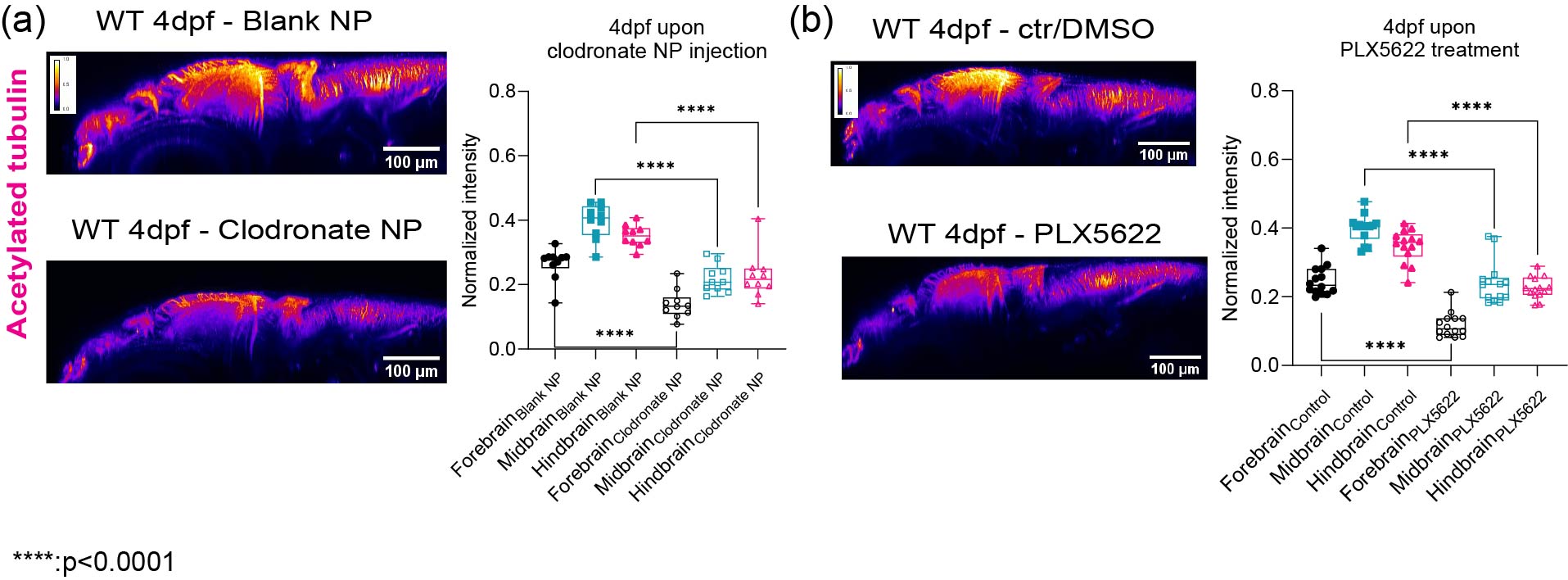


**Extended Data6:**

(a) Micrographs depicting the y-axis maximum intensity projection (xz) of wild type 4 dpf acetylated tubulin in terms of clodronate NP injection (b) Quantitation of normalized acetylated tubulin intensity in different brain regions upon clodronate NP injection (control NP: n = 12 and clodronate NP: n = 10). **** p<0.0001, unpaired two-tailed t-tests; confidence interval = 95%. (c) Micrographs depicting the y-axis maximum intensity projection (xz) of wild type 4 dpf acetylated tubulin for control/DMSO and PLX5622 treatment (b) Quantitation of normalized acetylated tubulin intensity for different brain regions upon PLX5622 treatment (control/DMSO: n = 14 and PLX5622: n = 13). **** p<0.0001, unpaired two-tailed t-tests; confidence interval = 95%.
